# Genotypic Characterization of* Rickettsia bellii* Reveals Distinct Lineages in the United States and South America

**DOI:** 10.1155/2018/8505483

**Published:** 2018-04-08

**Authors:** Felipe S. Krawczak, Marcelo B. Labruna, Joy A. Hecht, Christopher D. Paddock, Sandor E. Karpathy

**Affiliations:** ^1^Department of Preventive Veterinary Medicine and Animal Health, Faculty of Veterinary Medicine, University of São Paulo, São Paulo, SP, Brazil; ^2^Rickettsial Zoonoses Branch, National Center for Emerging and Zoonotic Infectious Diseases, Centers for Disease Control and Prevention, Atlanta, GA, USA

## Abstract

The bacterium* Rickettsia bellii* belongs to a basal group of rickettsiae that diverged prior to the pathogenic spotted fever group and typhus group* Rickettsia *species. Despite a diverse representation of* R. bellii* across more than 25 species of hard and soft ticks in the American continent, phylogeographical relationships among strains of this basal group-*Rickettsia* species are unknown; the work described here explores these relationships. DNA was extracted from 30* R. bellii *tick isolates: 15 from the United States, 14 from Brazil, and 1 from Argentina. A total of 2,269 aligned nucleotide sites of 3 protein coding genes (*glt*A,* atp*A, and* cox*A) and 2 intergenic regions (*rpm*E*-tRN*A^fmet^ and* RC1027-xth*A*2*) were concatenated and subjected to phylogenetic analysis by Bayesian methods. Results showed a separation of almost all isolates between North and South Americas, suggesting that they have radiated within their respective continents. Phylogenetic positions of the 30 isolates could be a result of not only their geographical origin but also the tick hosts they have coevolved with. Whether* R. bellii *originated with ticks in North or South America remains obscure, as our analyses did not show evidence for greater genetic divergence of* R. bellii *in either continent.

## 1. Introduction

Members of the genus* Rickettsia* (Rickettsiales: Rickettsiaceae) are Gram-negative obligate intracellular bacteria, usually in association with arthropods, although a number of distinct genotypes have also been described from leeches, amoeba, ciliate, and hydra [1]. Phylogenetic analysis-based studies have classified the* Rickettsia *species into five major groups, namely, the spotted fever group (SFG), the typhus group (TG), the transitional group (TRG), the* Rickettsia canadensis* group, and the* Rickettsia bellii* group [1, 2]. This later group occupies a basal position in all major phylogenetic studies, which indicates early divergence within the genus [1, 3, 4].

*Rickettsia bellii* was formally described in 1983 based on isolates obtained from multiple species of ixodids (hard ticks) and argasids (soft ticks) in the United States [5]. In the United States,* R. bellii *has been reported to be infecting the following 8 tick species:* Dermacentor variabilis, Dermacentor occidentalis, Dermacentor andersoni, Dermacentor albipictus, Dermacentor parumapertus, Haemaphysalis leporispalustris, Ornithodoros concanensis, *and* Argas cooleyi* [5]. In recent years, investigators in Latin America have identified* R. bellii *in ixodid ticks throughout Latin America, including El Salvador, Costa Rica, Panama, Colombia, Brazil, Peru, and Argentina [6–13]. In Central and South America,* R. bellii *has been detected in at least 19 tick species, namely,* Ixodes loricatus, Haemaphysalis juxtakochi, Amblyomma aureolatum, Amblyomma dubitatum, Amblyomma humerale, Amblyomma incisum, Amblyomma neumanni, Amblyomma longirostre, Amblyomma naponense, Amblyomma nodosum, Amblyomma ovale, Amblyomma oblongoguttatum, Amblyomma parvum, Amblyomma pseudoconcolor, Amblyomma rotundatum, Amblyomma sabanerae, Amblyomma scalpturatum, Amblyomma tigrinum*, and* Amblyomma varium* [6, 7, 12, 14–16].

The wide host range and extraordinarily broad distribution of this* Rickettsia* species are intriguing and although* R. bellii* is generally considered as nonpathogenic for animals and humans [2, 17], it could play an important role in the ecology and epidemiology of other pathogenic tick-borne SFG rickettsiae in the Americas [18]. Despite the diverse representation of* R. bellii* across more than 25 species of hard and soft ticks in the American continent, the phylogeographical relationships of this basal group-*Rickettsia* species has not been examined; the work described here explores these relationships, based on a hypothesis that* R. bellii *could have evolved firstly in one continent or a particular tick group and then radiated to other continents or other tick groups.

## 2. Materials and Methods

### 2.1. Rickettsial Isolates

**Figure 1:**
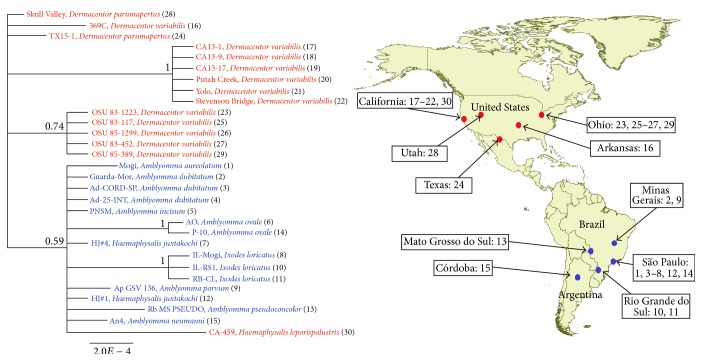
Molecular phylogenetic analysis of 30 isolates of* Rickettsia bellii* from North and South America.

*Rickettsia bellii* was isolated from various species of hard ticks collected in South America and North America, as described in the original publication of each of the isolates listed in Table 1. A total of 30 isolates of* R. bellii* from 13 species of hard ticks from 3 countries were used for the analysis (Figure 1). These included 1 from Argentina, 14 from Brazil, and 15 from USA (Table 1). The Brazilian and Argentinean isolates are available at the Rickettsial Collection of the Laboratory of Parasitic Diseases of Faculdade de Medicina Veterinária e Zootecnia (FMVZ) of the University of São Paulo (USP), and the US isolates are available at the Rickettsial Zoonoses Branch at the Centers for Disease Control and Prevention (CDC), Atlanta, Georgia, USA.

### 2.2. DNA Extraction and PCR

DNA from the* R. bellii* isolates from South America was extracted using the DNeasy Blood and Tissue Kit (Qiagen, Valencia, CA) and the QIAamp DNA Mini Kit (Qiagen) was used for the 15 isolates from North America, all in accordance with the manufacturer's recommendations. Amplification of fragments of five rickettsial genes and fifteen intergenic regions was attempted with the primer pairs listed in Table 2. Each PCR reaction consisted of 2 *μ*l template DNA, 20 picomoles of each primer, and 10 *μ*l Taq PCR Master Mix (Qiagen), while cycling conditions included a 1-minute incubation at 95°C followed by 35 cycles of a 30-second denaturation at 95°C, a 30-second annealing incubation (Table 2), and a 1-minute extension at 72°C. This was followed by a final 10-minute extension at 72°C. Gradient PCR was used to optimize the annealing temperatures in* R. bellii* for each primer pair.

### 2.3. DNA Purification and Sequencing

DNA fragments amplified by PCR were visualized using a UV lamp in a 1.5% agarose gel containing 0.1 *μ*g/ml ethidium bromide. PCR products of the appropriate size were cut from the gel and then purified using the Wizard SV gel and PCR clean-up system (Promega, Madison, WI). Sequencing reactions were prepared using one microliter of purified PCR product and the BigDye Terminator v3.1 Cycle Sequencing Kit (Applied Biosystems, Foster City, CA) according to the manufacturer's instructions and then sequenced using an ABI 3100 genetic analyzer (Applied Biosystems). Each PCR amplicon was sequenced at least once in both directions.

### 2.4. Sequence Alignment and Phylogenetic Inferences

The resulting sequences were assembled using Geneious® 9.1.4 software (Biomatters Ltd., Auckland, New Zealand) and aligned using ClustalW [35] and MEGA 6.0.6 software [36]. The resulting alignment was examined by eye to ensure proper alignment of the sequences and the simple indel-coding method [37] was used to remove insertions/deletions. MrBayes 3.2.6 [38] was used in Geneious® to perform a Bayesian phylogenetic analysis. The Jukes-Cantor model was used in an analysis consisting of 10,000,000 generations (1,000,000 burn-ins). The analysis included 3 heated chains, and an effective sample size (ESS) of 901 was achieved for all parameters.

### 2.5. Accession Numbers

The GenBank accession numbers for the DNA sequences generated in this study for the 30* R. bellii *isolates shown in Table 1 are the following:* glt*A gene (MF154866–MF154895),* atp*A gene (MF154926–MF154955),* cox*A gene (MF154896–MF154925),* rpm*E*-tRN*A^fmet^ intergenic region (MF154956–MF154985), and* RC1027-xth*A*2 *intergenic region (MF154986–MF155015).

## 3. Results

Among the 20 primer pairs used for amplification of rickettsial DNA fragments, only the primer pairs # 15, 16, 17, 19, and 20 (Table 2) were successful in amplifying DNA from the* R. bellii *isolates. These primers corresponded to 3 protein coding genes (*glt*A,* atp*A, and* cox*A) and 2 intergenic regions (*rpm*E*-tRN*A^fmet^ and RC1027*-xth*A*2*). The nucleotide sequences from the five loci for each of the 30 isolates were concatenated and used to perform a Bayesian analysis of their phylogeny. There was a separation of the isolates into at least three clades: one representing all South American isolates and the United States isolate CA-459 cultivated from a* H. leporispalustris* tick collected in northern California [39], one comprising 6 isolates from* D. variabilis* collected in northern California [29], and a third clade comprising 5 isolates from* D. variabilis* collected in Ohio [30] (Figure 1). North American isolates from* D. parumapertus *and the 369-C strain from* D. variabilis* did not group within any of the above clades. Within the South American clade, there were two smaller well-supported clades (each with a posterior probability of 1.0), one consisting of three isolates associated with* I. loricatus *ticks and one consisting of two* A. ovale*-associated isolates.

Overall, there were very few polymorphisms (<0.5%) between the 30* R. bellii *isolates. In the 2,269-nucleotide alignment, identity values between the isolates varied from 99.56 to 100% (Table S1). DNA sequences of the isolates composing the main South American clade were 99.69–100% identical and at the same time 99.56–99.74% identical to the clade containing* D. variabilis *isolates from California and 99.74–99.91% identical to the clade containing* D. variabilis *isolates from Ohio. Within-clade identities were 99.74–100% for the* D. variabilis *isolates from Ohio and 100% for the* D. variabilis *isolates from California; the Ohio clade was 99.74% identical to the California clade. Finally, sequences of the three North American isolates (two from* D. parumapertus *and one from* D. variabilis*) that did not group within any main clade were 99.87–99.96% identical to each other and 99.60–99.91% identical to the remaining isolates.

## 4. Discussion

Here we performed for the first time a phylogenetic analysis of multiple isolates of* R. bellii. *Because these isolates represented a number of different locations in North and South America, our intention was to infer phylogeographical relationships. Indeed, there was a separation of almost all isolates between the two continents, suggesting that they have radiated within their respective continents. Although the posterior probability of the separation of the South American clade from the North American isolates was not strong (PP = 0.59), this topology was also achieved using both the GTR and HKY85 evolutionary models (supplemental material), giving additional evidence for this separation. At the same time, it is noteworthy that while the North American clades represented isolates exclusively from* Dermacentor *ticks, the South American clade was composed of isolates from the genera* Amblyomma, Haemaphysalis, *and* Ixodes. *Under such circumstances, the phylogenetic positions of the 30 isolates of the present study could be a result of not only their geographical origin but also the tick hosts in which they were isolated from. This later assumption is corroborated by the position of the isolate from* H. leporispalustris*, which, despite being from North America, grouped within the South American clade, where at least two other isolates from* Haemaphysalis *ticks were present.

To our knowledge, the number and diversity of tick species infected with* R. bellii *are the largest and broadest described among species in the genus* Rickettsia*. The magnitude of different host species indicates horizontal transmission among tick populations, especially in South America, where isolates from different tick species and genera grouped together in the same clade. It is generally accepted that* R. bellii *is not pathogenic for vertebrates; in fact,* R. bellii *has never been isolated from a vertebrate host [2]. On the other hand, there has been serological evidence of animal natural infection or exposure by* R. bellii* [40, 41]. While* R. bellii *horizontal transmission among ticks via vertebrate host cannot be discarded, another likely mechanism could be via tick parasitoids, since the parasitism of the hymenopteran* Ixodiphagus *spp. is relatively common among different tick genera in the Americas [42, 43]. In fact, a recent study provided molecular detection of tick-borne rickettsiae in* Ixodiphagus *wasps that had emerged from ticks [44], highlighting the possibility that rickettsial organisms could be shared by ticks and their parasitoids. Regardless of the mechanisms of horizontal transmission, the tendency of each* R. bellii *genotype to be associated with a different tick species (Figure 1) suggests that horizontal transmission was more efficient at earlier times; thereafter, most of the* R. bellii *isolates are likely to have coevolved specifically with their specific tick species host, possibly towards a symbiotic association. In fact, analysis of the genome of the type strain of* R. bellii *has shown features compatible with various symbiotic bacteria, such as a large genome size with high coding capacity, in contrast to the reduced genome size with low coding capacity of the pathogenic rickettsiae [4, 45].

It has been proposed that the* R. bellii* group diverged prior to the division between the SFG and the TG, forming a basal group that also includes various herbivorous arthropod symbionts [1, 46]. While the genus* Rickettsia *was considered to be approximately 150 million years old, the divergence of the* R. bellii* group was estimated to have occurred much more recently, around 50 million years ago [1]. Analyses of the oldest tick fossils, from the Cretaceous, indicate that extant tick genera* (Amblyomma, Ornithodoros)* were soundly established around 100 million years ago [47–49]. From these data, it can be inferred that* R. bellii *likely radiated with its principal hosts approximately 50 million years ago. While ticks and rickettsiae are distributed in all continents, it is noteworthy that, under natural conditions and without human interference, tick species from the New World do not occur in the Old World and vice versa; the only exceptions are a few tick species that are associated with transcontinental marine birds [50]. Indeed, this scenario has accounted for the presence of* R. bellii-*infected ticks restricted to the New World, regardless of the ability of this bacterium to infect a vast array of tick genera and species. On the other hand, whether* R. bellii *originated with ticks in North or South America remains obscure, since our phylogenetic analyses did not show any evidence for greater genetic divergence of* R. bellii *in any of the two continents. Further analyses encompassing more* R. bellii *isolates from different tick genera and species, encompassing more molecular markers, should provide cues for the origin and radiation of* R. bellii *in the New World.

## 5. Conclusion 

Phylogeographical analysis of 30 strains (15 from North America and 15 from South America) isolated from 13 species of 4 genera shows a clear differentiation between most of the North and South American isolates, indicating geographic isolation between isolates of these two continents. Additionally, this analysis separated isolates of* R. bellii* by the species of tick from which these were isolated, indicating that the isolates could have coevolved with their tick vectors over time.

## Figures and Tables

**Table 1:** *Rickettsia bellii* isolates from South and North America used in the present study.

Number^*∗*^	Isolate name	Tick host	Geographic origin	Reference
(1)	Mogi	*Amblyomma aureolatum*	Mogi das Cruzes-SP, Brazil	[19]
(2)	Ad-MG	*Amblyomma dubitatum*	Guarda-Mor-MG, Brazil	[16]
(3)	Ad-CORD-SP	*A. dubitatum*	Cordeirópolis -SP, Brazil	[20]
(4)	Ad-25-INT	*A. dubitatum*	Ribeirão Grande-SP, Brazil	[20]
(5)	PNSM	*Amblyomma incisum*	Cubatão-SP, Brazil	[21]
(6)	AO	*Amblyomma ovale*	Ribeirão Grande-SP, Brazil	[22]
(7)	HJ#4	*Haemaphysalis juxtakochi*	Ribeirão Grande-SP, Brazil	[23]
(8)	IL-Mogi	*Ixodes loricatus*	Mogi das Cruzes-SP, Brazil	[24]
(9)	Ap GSV 136	*Amblyomma parvum*	Chapada Gaúcha-MG, Brazil	[25]
(10)	IL-RS1	*I. loricatus*	Derrubadas-RS, Brazil	[26]
(11)	RB-CL	*I. loricatus*	Cerro Largo-RS, Brazil	[13]
(12)	HJ#1	*H. juxtakochi*	São Paulo-SP, Brazil	[23]
(13)	Ap-MS	*Amblyomma pseudoconcolor*	Corumbá-MS, Brazil	[16]
(14)	P-10	*A. ovale*	Peruíbe-SP, Brazil	[27]
(15)	An4	*Amblyomma neumanni*	Deán Funes-Córdoba, Argentina	[28]
(16)	369-C	*Dermacentor variabilis*	Washington Co., Arkansas, USA	[5]
(17)	CA13-1	*Dermacentor variabilis*	Yolo Co., California, USA	[29]
(18)	CA13-9	*D. variabilis*	Yolo Co., California, USA	[29]
(19)	CA13-17	*D. variabilis*	Yolo Co., California, USA	[29]
(20)	Putah Creek	*D. variabilis*	Solano Co., California, USA	[29]
(21)	Yolo	*D. variabilis*	Yolo Co., California, USA	[29]
(22)	Stevenson Bridge	*D. variabilis*	Yolo Co., California, USA	[29]
(23)	OSU 83-1223	*D. variabilis*	Knox County, Ohio, USA	[30]
(24)	TX15-1	*Dermacentor parumapertus*	Brewster County, Texas, USA	[31]
(25)	OSU 83-117	*D. variabilis*	Licking County, Ohio, USA	[30]
(26)	OSU 85-1299	*D. variabilis*	Morrow County, Ohio, USA	[30]
(27)	OSU 83-452	*D. variabilis*	Coshocton County, Ohio, USA	[30]
(28)	Skull Valley	*D. parumapertus*	Tooele County, Utah, USA	[31]
(29)	OSU 85-389	*D. variabilis*	Ohio, USA	[30]
(30)	CA-459	*Haemaphysalis leporispalustris*	Mendocino Co., California, USA	[32]

^*∗*^These numbers are represented in Figure 1 as the geographical location of each isolate.

**Table 2:** Primer pairs used for amplification of rickettsial genes or intergenic regions in the present study.

Primer pair	Target	Forward primer (5′ to 3′)	Reverse primer (5′ to 3′)	Annealing temperature (°C)	Amplicon size (nt)	Reference
(1)	*mppA-purC*	GCAATTATCGGTCCGAATG	TTTCATTTATTTGTCTCAAAATTCA	45 to 55^1^	160	[33]
(2)	*nusG-rplK*	CAGTTGCAATATTGGTAAAGCA	CAGCAGCTGGAATTATCAAGTT	45 to 55^1^	270	[33]
(3)	*murG-RC0563*	GAAGAAAAGAAGGGCATAAGCTA	CAAGCTGAAAGTAAAAACATTCC	40 to 52^1^	293	[33]
(4)	*ntrY-rpsU*	AGCTGCTGTTGCTAAAGTAAAAA	CAAGAAGCAGCAAGAAGACAGA	52 to 64^1^	363	[33]
(5)	*dksA-xerC*	TCCCATAGGTAATTTAGGTGTTTC	TACTACCGCATATCCAATTAAAAA	45 to 55^1^	416	[33]
(6)	*spo0J-abcT1*	AAAGATTTGGAAGAATTAGACTTGAT	TTTGCTTAAACCAACCATTTCA	45 to 55^1^	259	[33]
(7)	*fabZ-lpxD*	TGTTAGGATCGATTTTAAGTACTCTATCT	TGGATTGGCATAGACAATCTATTA	45 to 55^1^	195	[33]
(8)	RC1137-*tlc-5*	CGGGATAACGCCGAGTAATA	ATGCCGCTCTGAATTTGTTT	45 to 55^1^	264	[33]
(9)	RC0230-RC0231	TGCACCCGCCTAAAACTAAC	ATGGTCGGCCGTAGAAAAA	45 to 55^1^	232	[33]
(10)	*groES*-RC0970	CTTGCATCGGCTTTTCTTTT	AGCTTTGAGCTGATGGGCTA	45 to 55^1^	215	[33]
(11)	*rrf-pyrH*	GAGCTTTCTCCATCTTTTCTTG	AAAGGGGAATATACGACAATTGAG	45 to 55^1^	238	[33]
(12)	tRNA^Gly^-tRNA^Tyr^	AGCTTGGAAGGCTGGAACTC	ATCCTTCTCCCTCCACCACT	45 to 55^1^	148	[33]
(13)	*pal*-RC1201	TGCAAGCACACATAATGCAA	TCAAAATCGATTCCTCTTTTCC	45 to 55^1^	216	[33]
(14)	*atpA*	ATCAAGCGTTGCACAGATAG	GGAAGTGCCGTAAGTGAACC	58	Unknown^*∗*^	[1]
(15)	*rpmE*-tRNA^fMet^	TTCCGGAAATGTAGTAAATCAATC	TCAGGTTATGAGCCTGACGA	54	144	[33]
(16)	*RC1027*-*xthA2*	GGTATGTAAATGAGCCTTATCAATACT	TCAGTAGTATAAGTAGCTCCTGCTGTC	54	351	[33]
(17)	*gltA*	GTCTACTGCTTCGTGTAGATCAAC	GGCTGACCTATAGAATATTTATAAGAC	54	408	[25]
(18)	*coxA*	ACAGCCGTTGATATGGCTA	CATATTCCAACCGGCAAAAG	58	Unknown^*∗*^	[1]
(19)	*coxA*	GGTGCTCCTGATATGGCATT	CATATTCCAGCCGGCAAAAG	58	Unknown^*∗*^	[1]
(20)	*atpA*	ATCAAGCGTTGCACAGATAG	CGACTTACCGAAATACCGAC	56	449	[1, 34]

^*∗*^Data not given in the original publication; ^1^tested in gradient PCR.

## Supplementary Materials

Supplementary Materials An identity matrix table of the 2,269-nucleotide alignment used for the phylogeny if* R. bellii *is available in the supplemental material for this article (Table S1). The final alignment comprising 2,269 nucleotides was concatenated in the following order:* rpm*E*-tRN*A^fmet^ (414-nt),* glt*A (357-nt),* cox*A (898-nt),* atp*A (449-nt), and RC1027*-xth*A2 (151-nt); and it is also available in the supplementary material (Rbellii final alignment.fas). Two additional phylogenetic trees are also available in the supplementary material (Rbellii GTR tree.pdf and Rbellii HKY tree.pdf). Table S1: identity matrix of a 2,269-nucleotide alignment of the 30* Rickettsia bellii* isolates from Brazil (BRA), Argentina (ARG), and the United States (USA) used in the present study. Supplementary Figure 1: molecular phylogenetic analysis of 30 isolates of* Rickettsia bellii* from North and South America. A total of 2,269 aligned nucleotide sites of 3 protein coding genes* (gltA*,* atpA*, and* coxA)* and 2 intergenic regions (*rpm*E-tRNA*fmet* and RC1027-*xth*A2) were concatenated and subjected to Bayesian analysis. A total of 10,000,000 generations were run using the GTR model with a sample frequency of 10,000. The analysis was run with 3 heated chains, and the first 1,000,000 generations were discarded as burn-in. Numbers at nodes are support values derived from posterior probability. The scale bar is in units of expected substitutions per site. Supplementary Figure 2: molecular phylogenetic analysis of 30 isolates of* Rickettsia bellii* from North and South America. A total of 2,269 aligned nucleotide sites of 3 protein coding genes* (gltA*,* atpA*, and* coxA)* and 2 intergenic regions (*rpm*E-tRNA*fmet* and RC1027-*xth*A2) were concatenated and subjected to Bayesian analysis. A total of 10,000,000 generations were run using the HKY85 model with a sample frequency of 10,000. The analysis was run with 3 heated chains, and the first 1,000,000 generations were discarded as burn-in. Numbers at nodes are support values derived from posterior probability. The scale bar is in units of expected substitutions per site.
