# Supplementary material for: Genotypic Characterization of Rickettsia bellii Reveals Distinct Lineages in the United States and South America
Source: Biomed Res Int. 2018 Apr 8;2018:8505483. doi: 10.1155/2018/8505483 (PMC5911327; doi:10.1155/2018/8505483)
Supplement: Supplementary Materials — An identity matrix table of the 2,269-nucleotide alignment used for the phylogeny if R. bellii is available in the supplemental material for this article (Table S1). The final alignment comprising 2,269 nucleotides was concatenated in the following order: rpmE-tRNAfmet (414-nt), gltA (357-nt), coxA (898-nt), atpA (449-nt), and RC1027-xthA2 (151-nt); and it is also available in the supplementary material (Rbellii final alignment.fas). Two additional phylogenetic trees are also available in the supplementary material (Rbellii GTR tree.pdf and Rbellii HKY tree.pdf). Table S1: identity matrix of a 2,269-nucleotide alignment of the 30 Rickettsia bellii isolates from Brazil (BRA), Argentina (ARG), and the United States (USA) used in the present study. Supplementary Figure 1: molecular phylogenetic analysis of 30 isolates of Rickettsia bellii from North and South America. A total of 2,269 aligned nucleotide sites of 3 protein coding genes (gltA, atpA, and coxA) and 2 intergenic regions (rpmE-tRNAfmet and RC1027-xthA2) were concatenated and subjected to Bayesian analysis. A total of 10,000,000 generations were run using the GTR model with a sample frequency of 10,000. The analysis was run with 3 heated chains, and the first 1,000,000 generations were discarded as burn-in. Numbers at nodes are support values derived from posterior probability. The scale bar is in units of expected substitutions per site. Supplementary Figure 2: molecular phylogenetic analysis of 30 isolates of Rickettsia bellii from North and South America. A total of 2,269 aligned nucleotide sites of 3 protein coding genes (gltA, atpA, and coxA) and 2 intergenic regions (rpmE-tRNAfmet and RC1027-xthA2) were concatenated and subjected to Bayesian analysis. A total of 10,000,000 generations were run using the HKY85 model with a sample frequency of 10,000. The analysis was run with 3 heated chains, and the first 1,000,000 generations were discarded as burn-in. Numbers at nodes are support values derived from p [file 8505483.f1.zip › Supplemental material_BMRI_2216106.pdf]

Rbellii final alignment
>Mogi-_Amblyomma_aureolatum_BRA
TAACAAAAGCATTAGTGATTTTAATAAAAGATTTTCCGGTCTTTCTTTCGGTAGTCAAAAAAAGGAAGCTAGTTAATTTCTATA
ATCGGGTGGAGACATTTCCGCATAGATCAAGAACGTTTCCAGTGTGTCATCACGTTGGCGGCTTTTTTACGTGGATCGAGAGTC
GTCATTGCGAGTGAATGAAATGAGCGTGGCAATCCAGAAAATAATAAAAAAATACTAATTTTATTAGTATTTTTAATTGGATCCC
CTGAATAAATCACGGGATGATAGGGGGAGAATGATCCACGTAACACACATTACTATGGGGTAAAAACTTACTCTAATCATTTAT
GTCGCTAATCTTTGTTTATAATATTAATAAACAAAGTCATCAAATTTCTTTATTGCGGGGTGGAGCAGCTCGGTAGCTATAACT
TATATAGATGGTGATCAAGGAATTTTGCGGCATCGTGGTTATGATATAAAAGATTTAGCCGAAAAAAGCGACTTTTTAGAGGTG
GCATATTTATTGATTTATGGGGAATTACCAAATAATAAGCAGTATAATGATTTTACTAAAAAGGTTGCTCATCATGCGTTAGTT
AATGAAAGATTACATTATTTATTCCAAACGTTTTGTAGCTCTTCGCATCCTATGGCTATTATGCTTGCGGCGGTTGGTTCTCTT
TCTGCATTTTATCCTGATTTGCTGAATTTTTTTAAAGAAGCGGATTATGAACTTACAGCTATTAGAATGATAGCTAAAATACCA
ACTATTGCTGCAATGGACACTCTACCCTCCTTTAAGTAGTATAAGCGGACATCCAGGAGCAGCGGTTGATATGGCTATTTTCAG
TCTGCATTTAACCGGTCTTTCATCAATACTTGGCTCAATCAACTTAATCGTTACTATCTTTAATATGAGAGCACCCGGGATGGG
ACTATTCAAAATGCCGTTATTTGTCTGGTCTATTTTAGTTACTGCATTCTTGATAATTTTAGCTATGCCAGTGCTTGGCGGAGC
TATTACTATGTTACTTACCGATCGTAATTTCGGTACTACTTTCTTTAAAACTGATGGTGGTGGTGATCCAGTATTATTTCAGCA
CTTATTTTGGTTTTTTGGTCACCCTGAAGTATATATTGTAATACTTCCAGGTTTTGGTATTGTAAGCCAAGTTATTTCCACTTT
CTCACGTAAACCAATATTTGGCTATCAAGGCATGGTTGGAGCCATGGTAATAATCGGCTTTGTCGGGTTTATTGTATGGGCTCA
CCATATGTTTACAGTTGGGCTTTCTTACAACGCACTTATATATTTTACTGCCGGAACAATGATTATCGCAATTCCAACAGGTAT
CAAAATATTTAGCTGGATCGCAACTATGTGGGGTGGATCGATTACTTTCCCAACGCCTATGCTATTCTCTATAGGATTTATTAT
ATTATTCACGATTGGCGGCGTAACTGGCATAATCTTATCAAACTCGGCACTTGATAGAGTTCTGCACGATACATATTATGTTGT
GGCACATTTCCATTATACGATGTCGCTCGGTGCTTTATTCACTGCATTTGCCGGCTTTTATTATTGGTTCGGTAAAATATCAGG
CAAGCAATATCCCGAAATCTTAGGCAAAATCCATTTCTGGATTACTTTTGTCGGTGTTAATCTAACTTTCTTTAAAAAAGCTGG
AAGATGCTGGGGCAATGGATTATACAATCATTGTTTCGGCTACTGCATCGGAAGCTGCTGCATTACAATTTGTTGCTCCTTATG
CCGCATGTAGTATGGGTGAGTATTTCCGTAATAACGGCAAGCATGCACTTATTATTTATGATGATTTAAGTAAACATGCTGTCG
CATATAGACAAATTTCATTGTTACTTAGAAGACCGCCCGGACGTGAAGCATATCCCGGTGACGTATTTTACTTGCATTCAAGAT
TACTTGAGTGTGCTGCTAAAATGTCAGAGGAGAAAGGCGGCGGTTCACTTACGGCACTTCCTATAATCGAAACCCAAGCAGGTG
ACGTATCTGCTTATATTCCAACAAACGTTATTTCTATTACTGACGGTCAAATTTTCTTAGAAAGCGAGCTGTTTTATAAAGGTA
TAAGACCGGCTGTTAATGGATGGTTACTTCAAATGGTAGTGCTAATGTTTCTGGTATGGATATTGAATTAATTAATGCTCTTGC
TGCAAAAATAGGTATTAATATCGAGTACCACCAAGATAATTGGTATCAAGATCAGTTAGATATTCAAAGCGGTGCTGCTGACAT
>Guarda_Mor-_Amblyomma_dubitatum_BRA
TAACAAAAGCATTAGTGATTTTAATAAAAGATTTTCCGGTCTTTCTTTCGGTAGTCAAAAAAAGGAAGCTAGTTAATTTCTATA
ATCGGGTGGAGACATTTCCGCATAGATCAAGAACGTTTCCAGTGTGTCATCACGTTGGCGGCTTTTTTACGTGGATCGAGAGTC
GTCATTGCGAGTGAATGAAATGAGCGTGGCAATCCAGAAAATAATAAAAAAATACTAATTTTATTAGTATTTTTAATTGGATCCC
CTGAATAAATCACGGGATGATAGGGGGAGAATGATCCACGTAACACACATTACTATGGGGTAAAAACTTACTCTAATCATTTAT
GTCGCTAATCTTTGTTTATAATATTAATAAACAAAGTCATCAAATTTCTTTATTGCGGGGTGGAGCAGCTCGGTAGCTATAACT
TATATAGATGGTGATCAAGGAATTTTGCGGCATCGTGGTTATGATATAAAAGATTTAGCCGAAAAAAGCGACTTTTTAGAGGTG
GCATATTTATTGATTTATGGGGAATTACCAAATAATAAGCAGTATAATGATTTTACTAAAAAGGTTGCTCATCATGCGTTAGTT
AATGAAAGATTACATTATTTATTCCAAACGTTTTGTAGCTCTTCGCATCCTATGGCTATTATGCTTGCGGCGGTTGGTTCTCTT
TCTGCATTTTATCCTGATTTGCTGAATTTTTTTAAAGAAGCGGATTATGAACTTACAGCTATTAGAATGATAGCTAAAATACCA
ACTATTGCTGCAATGGACACTCTACCCTCCTTTAAGTAGTATAAGCGGACATCCAGGAGCAGCGGTTGATATGGCTATTTTCAG
TCTGCATTTAACCGGTCTTTCATCAATACTTGGCTCAATCAACTTAATCGTTACTATCTTTAATATGAGAGCACCCGGGATGGG
ACTATTCAAAATGCCGTTATTTGTCTGGTCTATTTTAGTTACTGCATTCTTGATAATTTTAGCTATGCCAGTGCTTGGCGGAGC
TATTACTATGTTACTTACCGATCGTAATTTCGGTACTACTTTCTTTAAAACTGATGGTGGTGGTGATCCAGTATTATTTCAGCA
CTTATTTTGGTTTTTTGGTCACCCTGAAGTATATATTGTAATACTTCCAGGTTTTGGTATTGTAAGCCAAGTTATTTCCACTTT
CTCACGTAAACCAATATTTGGCTATCAAGGCATGGTTGGAGCCATGGTAATAATCGGCTTTGTCGGGTTTATTGTATGGGCTCA
CCATATGTTTACAGTTGGGCTTTCTTACAACGCACTTATATATTTTACTGCCGGAACAATGATTATCGCAATTCCAACAGGTAT
CAAAATATTTAGCTGGATCGCAACTATGTGGGGTGGATCGATTACTTTCCCAACGCCTATGCTATTCTCTATAGGATTTATTAT
ATTATTCACGATTGGCGGCGTAACTGGCATAATCTTATCAAACTCGGCACTTGATAGAGTTCTGCACGATACATATTATGTTGT
GGCACATTTCCATTATACGATGTCGCTCGGTGCTTTATTCACTGCATTTGCCGGCTTTTATTATTGGTTCGGTAAAATATCAGG
CAAGCAATATCCCGAAATCTTAGGCAAAATCCATTTCTGGATTACTTTTGTCGGTGTTAATCTAACTTTCTTTAAAAAAGCTGG
AAGATGCTGGGGCAATGGATTATACAATCATTGTTTCGGCTACTGCATCGGAAGCTGCTGCATTACAATTTGTTGCTCCTTATG
CCGCATGTAGTATGGGTGAGTATTTCCGTGATAACGGCAAGCATGCACTTATTATTTATGATGATTTAAGTAAACATGCTGTCG
CATATAGACAAATTTCATTGTTACTTAGAAGACCGCCCGGACGTGAAGCATATCCCGGTGACGTATTTTACTTGCATTCAAGAT
TACTTGAGTGTGCTGCTAAAATGTCAGAGGAGAAAGGCGGCGGTTCACTTACGGCACTTCCTATAATCGAAACCCAAGCAGGTG
ACGTATCTGCTTATATTCCAACAAACGTTATTTCTATTACTGACGGTCAAATTTTCTTAGAAAGCGAGCTGTTTTATAAAGGTA
TAAGACCGGCTGTTAATGGATGGTTACTTCAAATGGTAGTGCTAATGTTTCTGGTATGGATATTGAATTAATTAATGCTCTTGC
TGCAAAAATAGGTATTAATATCGAGTACCACCAAGATAATTGGTATCAAGATCAGTTAGATATTCAAAGCGGTGCTGCTGACAT
>Cord-_Amblyomma_dubitatum_BRA
TAACAAAAGCATTAGTGATTTTAATAAAAGATTTTCCGGTCTTTCTTTCGGTAGTCAAAAAAAGGAAGCTAGTTAATTTCTATA
ATCGGGTGGAGACATTTCCGCATAGATCAAGAACGTTTCCAGTGTGTCATCACGTTGGCGGCTTTTTTACGTGGATCGAGAGTC
GTCATTGCGAGTGAATGAAATGAGCGTGGCAATCCAGAAAATAATAAAAAAATACTAATTTTATTAGTATTTTTAATTGGATCCC
CTGAATAAATCACGGGATGATAGGGGGAGAATGATCCACGTAACACACATTACTATGGGGTAAAAACTTACTCTAATCATTTAT
GTCGCTAATCTTTGTTTATAATATTAATAAACAAAGTCATCAAATTTCTTTATTGCGGGGTGGAGCAGCTCGGTAGCTATAACT
TATATAGATGGTGATCAAGGAATTTTGCGGCATCGTGGTTATGATATAAAAGATTTAGCCGAAAAAAGCGACTTTTTAGAGGTG

GCATATTTATTGATTTATGGGGAATTACCAAATAATAAGCAGTATAATGATTTTACTAAAAAGGTTGCTCATCATGCGTTAGTT
AATGAAAGATTACATTATTTATTCCAAACGTTTTGTAGCTCTTCGCATCCTATGGCTATTATGCTTGCGGCGGTTGGTTCTCTT
TCTGCATTTTATCCTGATTTGCTGAATTTTTTTAAAGAAGCGGATTATGAACTTACAGCTATTAGAATGATAGCTAAAATACCA
ACTATTGCTGCAATGGACACTCTACCCTCCTTTAAGTAGTATAAGCGGACATCCAGGAGCAGCGGTTGATATGGCTATTTTCAG
TCTGCATTTAACCGGTCTTTCATCAATACTTGGCTCAATCAACTTAATCGTTACTATCTTTAATATGAGAGCACCCGGGATGGG
ACTATTCAAAATGCCGTTATTTGTCTGGTCTATTTTAGTTACTGCATTCTTGATAATTTTAGCTATGCCAGTGCTTGGCGGAGC
TATTACTATGTTACTTACCGATCGTAATTTCGGTACTACTTTCTTTAAAACTGATGGTGGTGGTGATCCAGTATTATTTCAGCA
CTTATTTTGGTTTTTTGGTCACCCTGAAGTATATATTGTAATACTTCCAGGTTTTGGTATTGTAAGCCAAGTTATTTCCACTTT
CTCACGTAAACCAATATTTGGCTATCAAGGCATGGTTGGAGCCATGGTAATAATCGGCTTTGTCGGGTTTATTGTATGGGCTCA
CCATATGTTTACAGTTGGGCTTTCTTACAACGCACTTATATATTTTACTGCCGGAACAATGATTATCGCAATTCCAACAGGTAT
CAAAATATTTAGCTGGATCGCAACTATGTGGGGTGGATCGATTACTTTCCCAACGCCTATGCTATTCTCTATAGGATTTATTAT
ATTATTCACGATTGGCGGCGTAACTGGCATAATCTTATCAAACTCGGCACTTGATAGAGTTCTGCACGATACATATTATGTTGT
GGCACATTTCCATTATACGATGTCGCTCGGTGCTTTATTCACTGCATTTGCCGGCTTTTATTATTGGTTCGGTAAAATATCAGG
CAAGCAATATCCCGAAATCTTAGGCAAAATCCATTTCTGGATTACTTTTGTCGGTGTTAATCTAACTTTCTTTAAAAAAGCTGG
AAGATGCTGGGGCAATGGATTATACAATCATTGTTTCGGCTACTGCATCGGAAGCTGCTGCATTACAATTTGTTGCTCCTTATG
CCGCATGTAGTATGGGTGAGTATTTCCGTGATAACGGCAAGCATGCACTTATTATTTATGATGATTTAAGTAAACATGCTGTCG
CATATAGACAAATTTCATTGTTACTTAGAAGACCGCCCGGACGTGAAGCATATCCCGGTGACGTATTTTACTTGCATTCAAGAT
TACTTGAGTGTGCTGCTAAAATGTCAGAGGAGAAAGGCGGCGGTTCACTTACGGCACTTCCTATAATCGAAACCCAAGCAGGTG
ACGTATCTGCTTATATTCCAACAAACGTTATTTCTATTACTGACGGTCAAATTTTCTTAGAAAGCGAGCTGTTTTATAAAGGTA
TAAGACCGGCTGTTAATGGATGGTTACTTCAAATGGTAGTGCTAATGTTTCTGGTATGGATATTGAATTAATTAATGCTCTTGC
TGCAAAAATAGGTATTAATATCGAGTACCACCAAGATAATTGGTATCAAGATCAGTTAGATATTCAAAGCGGTGCTGCTGACAT
>Ad_25-_Amblyomma_dubitatum_BRA
TAACAAAAGCATTAGTGATTTTAATAAAAGATTTTCCGGTCTTTCTTTCGGTAGTCAAAAAAAGGAAGCTAGTTAATTTCTATA
ATCGGGTGGAGACATTTCCGCATAGATCAAGAACGTTTCCAGTGTGTCATCACGTTGGCGGCTTTTTTACGTGGATCGAGAGTC
GTCATTGCGAGTGAATGAAATGAGCGTGGCAATCCAGAAAATAATAAAAAATACTAATTTTATTAGTATTTTTAATTGGATCCC
CTGAATAAATCACGGGATGATAGGGGGAGAATGATCCACGTAACACACATTACTATGGGGTAAAAACTTACTCTAATCATTTAT
GTCGCTAATCTTTGTTTATAATATTAATAAACAAAGTCATCAAATTTCTTTATTGCGGGGTGGAGCAGCTCGGTAGCTATAACT
TATATAGATGGTGATCAAGGAATTTTGCGGCATCGTGGTTATGATATAAAAGATTTAGCCGAAAAAAGCGACTTTTTAGAGGTG
GCATATTTATTGATTTATGGGGAATTACCAAATAATAAGCAGTATAATGATTTTACTAAAAAGGTTGCTCATCATGCGTTAGTT
AATGAAAGATTACATTATTTATTCCAAACGTTTTGTAGCTCTTCGCATCCTATGGCTATTATGCTTGCGGCGGTTGGTTCTCTT
TCTGCATTTTATCCTGATTTGCTGAATTTTTTTAAAGAAGCGGATTATGAACTTACAGCTATTAGAATGATAGCTAAAATACCA
ACTATTGCTGCAATGGACACTCTACCCTCCTTTAAGTAGTATAAGCGGACATCCAGGAGCAGCGGTTGATATGGCTATTTTCAG
TCTGCATTTAACCGGTCTTTCATCAATACTTGGCTCAATCAACTTAATCGTTACTATCTTTAATATGAGAGCACCCGGGATGGG
ACTATTCAAAATGCCGTTATTTGTCTGGTCTATTTTAGTTACTGCATTCTTGATAATTTTAGCTATGCCAGTGCTTGGCGGAGC
TATTACTATGTTACTTACCGATCGTAATTTCGGTACTACTTTCTTTAAAACTGATGGTGGTGGTGATCCAGTATTATTTCAGCA
CTTATTTTGGTTTTTTGGTCACCCTGAAGTATATATTGTAATACTTCCAGGTTTTGGTATTGTAAGCCAAGTTATTTCCACTTT
CTCACGTAAACCAATATTTGGCTATCAAGGCATGGTTGGAGCCATGGTAATAATCGGCTTTGTCGGGTTTATTGTATGGGCTCA
CCATATGTTTACAGTTGGGCTTTCTTACAACGCACTTATATATTTTACTGCCGGAACAATGATTATCGCAATTCCAACAGGTAT
CAAAATATTTAGCTGGATCGCAACTATGTGGGGTGGATCGATTACTTTCCCAACGCCTATGCTATTCTCTATAGGATTTATTAT
ATTATTCACGATTGGCGGCGTAACTGGCATAATCTTATCAAACTCGGCACTTGATAGAGTTCTGCACGATACATATTATGTTGT
GGCACATTTCCATTATACGATGTCGCTCGGTGCTTTATTCACTGCATTTGCCGGCTTTTATTATTGGTTCGGTAAAATATCAGG
CAAGCAATATCCCGAAATCTTAGGCAAAATCCATTTCTGGATTACTTTTGTCGGTGTTAATCTAACTTTCTTTAAAAAAGCTGG
AAGATGCTGGGGCAATGGATTATACAATCATTGTTTCGGCTACTGCATCGGAAGCTGCTGCATTACAATTTGTTGCTCCTTATG
CCGCATGTAGTATGGGTGAGTATTTCCGTGATAACGGCAAGCATGCACTTATTATTTATGATGATTTAAGTAAACATGCTGTCG
CATATAGACAAATTTCATTGTTACTTAGAAGACCGCCCGGACGTGAAGCATATCCCGGTGACGTATTTTACTTGCATTCAAGAT
TACTTGAGTGTGCTGCTAAAATGTCAGAGGAGAAAGGCGGCGGTTCACTTACGGCACTTCCTATAATCGAAACCCAAGCAGGTG
ACGTATCTGCTTATATTCCAACAAACGTTATTTCTATTACTGACGGTCAAATTTTCTTAGAAAGCGAGCTGTTTTATAAAGGTA
TAAGACCGGCTGTTAATGGATGGTTACTTCAAATGGTAGTGCTAATGTTTCTGGTATGGATATTGAATTAATTAATGCTCTTGC
TGCAAAAATAGGTATTAATATCGAGTACCACCAAGATAATTGGTATCAAGATCAGTTAGATATTCAAAGCGGTGCTGCTGACAT
>PNSM-_Amblyomma_incisum_BRA
TAACAAAAGCATTAGTGATTTTAATAAAAGATTTTCCGGTCTTTCTTTCGGTAGTCAAAAAAAGGAAGCTAGTTAATTTCTATA
ATCGGGTGGAGACATTTCCGCATAGATCAAGAACGTTTCCAGTGTGTCATCACGTTGGCGGCTTTTTTACGTGGATCGAGAGTC
GTCATTGCGAGTGAATGAAATGAGCGTGGCAATCCAGAAAATAATAAAAAATACTAATTTTATTAGTATTTTTAATTGGATCCC
CTGAATAAATCACGGGATGATAGGGGGAGAATGATCCACGTAACACACATTACTATGGGGTAAAAACTTACTCTAATCATTTAT
GTCGCTAATCTTTGTTTATAATATTAATAAACAAAGTCATCAAATTTCTTTATTGCGGGGTGGAGCAGCTCGGTAGCTATAACT
TATATAGATGGTGATCAAGGAATTTTGCGGCATCGTGGTTATGATATAAAAGATTTAGCCGAAAAAAGCGACTTTTTAGAGGTG
GCATATTTATTGATTTATGGGGAATTACCAAATAATAAGCAGTATAATGATTTTACTAAAAAGGTTGCTCATCATGCGTTAGTT
AATGAAAGATTACATTATTTATTCCAAACGTTTTGTAGCTCTTCGCATCCTATGGCTATTATGCTTGCGGCGGTTGGTTCTCTT
TCTGCATTTTATCCTGATTTGCTGAATTTTTTTAAAGAAGCGGATTATGAACTTACAGCTATTAGAATGATAGCTAAAATACCA
ACTATTGCTGCAATGGACACTCTACCCTCCTTTAAGTAGTATAAGCGGACATCCAGGAGCAGCGGTTGATATGGCTATTTTCAG
TCTGCATTTAACCGGTCTTTCATCAATACTTGGCTCAATCAACTTAATCGTTACTATCTTTAATATGAGAGCACCCGGGATGGG
ACTATTCAAAATGCCGTTATTTGTCTGGTCTATTTTAGTTACTGCATTCTTGATAATTTTAGCTATGCCAGTGCTTGGCGGAGC
TATTACTATGTTACTTACCGATCGTAATTTCGGTACTACTTTCTTTAAAACTGATGGTGGTGGTGATCCAGTATTATTTCAGCA

CTTATTTTGGTTTTTTGGTCACCCTGAAGTATATATTGTAATACTTCCAGGTTTTGGTATTGTAAGCCAAGTTATTTCCACTTT
CTCACGTAAACCAATATTTGGCTATCAAGGCATGGTTGGAGCCATGGTAATAATCGGCTTTGTCGGGTTTATTGTATGGGCTCA
CCATATGTTTACAGTTGGGCTTTCTTACAACGCACTTATATATTTTACTGCCGGAACAATGATTATCGCAATTCCAACAGGTAT
CAAAATATTTAGCTGGATCGCAACTATGTGGGGTGGATCGATTACTTTCCCAACGCCTATGCTATTCTCTATAGGATTTATTAT
ATTATTCACGATTGGCGGCGTAACTGGCATAATCTTATCAAACTCGGCACTTGATAGAGTTCTGCACGATACATATTATGTTGT
GGCACATTTCCATTATACGATGTCGCTCGGTGCTTTATTCACTGCATTTGCCGGCTTTTATTATTGGTTCGGTAAAATATCAGG
CAAGCAATATCCCGAAATCTTAGGCAAAATCCATTTCTGGATTACTTTTGTCGGTGTTAATCTAACTTTCTTTAAAAAAGCTGG
AAGATGCTGGGGCAATGGATTATACAATCATTGTTTCGGCTACTGCATCGGAAGCTGCTGCATTACAATTTGTTGCTCCTTATG
CCGCATGTAGTATGGGTGAGTATTTCCGTGATAACGGCAAGCATGCACTTATTATTTATGATGATTTAAGTAAACATGCTGTCG
CATATAGACAAATTTCATTGTTACTTAGAAGACCGCCCGGACGTGAAGCATATCCCGGTGACGTATTTTACTTGCATTCAAGAT
TACTTGAGTGTGCTGCTAAAATGTCAGAGGAGAAAGGCGGCGGTTCACTTACGGCACTTCCTATAATCGAAACCCAAGCAGGTG
ACGTATCTGCTTATATTCCAACAAACGTTATTTCTATTACTGACGGTCAAATTTTCTTAGAAAGCGAGCTGTTTTATAAAGGTA
TAAGACCGGCTGTTAATGGATGGTTACTTCAAATGGTAGTGCTAATGTTTCTGGTATGGATATTGAATTAATTAATGCTCTTGC
TGCAAAAATAGGTATTAATATCGAGTACCACCAAGATAATTGGTATCAAGATCAGTTAGATATTCAAAGCGGTGCTGCTGACAT
>AO-_Amblyomma_ovale_BRA
TAACAAAAGCATTAGTGATTTTAATAAAAGATTTTCCGGTCTTTCTTTCGGTAGTCAAAAAAAGGAAGCTAGTTAATTTCTATA
ATCGGGTGGAGACATTTCCGCATAGATCAAGAACGTTTCCAGTGTGTCATCACGTTGGCGGCTTTTTTACGTGGATCGAGAGTC
GTCATTGCGAGTGAATGAAATGAGCGTGGCAATCCAGAAAATAATAAAAAATACTAATTTTATTAGTATTTTTAATTGGATCCC
TTGAATAAATCACGGGATGATAGGGGGAGAATGATCCACGTAACACACATTACTATGGGGTAAAAACTTACTCTAATCATTTAT
GTCGCTAATCTTTGTTTATAATATTAATAAACAAAGTCATCAAATTTCTTTATTGCGGGGTGGAGCAGCTCGGTAGCTATAACT
TATATAGATGGTGATCAAGGAATTTTGCGGCATCGTGGTTATGATATAAAAGATTTAGCCGAAAAAAGCGACTTTTTAGAGGTG
GCATATTTATTGATTTATGGGGAATTACCAAATAATAAGCAGTATAATGATTTTACTAAAAAGGTTGCTCATCATGCGTTAGTT
AATGAAAGATTACATTATTTATTCCAAACGTTTTGTAGCTCTTCGCATCCTATGGCTATTATGCTTGCGGCGGTTGGTTCTCTT
TCTGCATTTTATCCTGATTTGCTGAATTTTTTTAAAGAAGCGGATTATGAACTTACAGCTATTAGAATGATAGCTAAAATACCA
ACTATTGCTGCAATGGACACTCTACCCTCCTTTAAGTAGTATAAGCGGACATCCAGGAGCAGCGGTTGATATGGCTATTTTCAG
TCTGCATTTAACCGGTCTTTCATCAATACTTGGCTCAATCAACTTAATCGTTACTATCTTTAATATGAGAGCACCCGGGATGGG
ACTATTCAAAATGCCGTTATTTGTCTGGTCTATTTTAGTTACTGCATTCTTGATAATTTTAGCTATGCCAGTGCTTGGCGGAGC
TATTACTATGTTACTTACCGATCGTAATTTCGGTACTACTTTCTTTAAAACTGATGGTGGTGGTGATCCAGTATTATTTCAGCA
CTTATTTTGGTTTTTTGGTCACCCTGAAGTATATATTGTAATACTTCCAGGTTTTGGTATTGTAAGCCAAGTTATTTCCACTTT
CTCACGTAAACCAATATTTGGCTATCAAGGCATGGTTGGAGCCATGGTAATAATCGGCTTTGTCGGGTTTATTGTATGGGCTCA
CCATATGTTTACAGTTGGGCTTTCTTACAACGCACTTATATATTTTACTGCCGGAACAATGATTATCGCAATTCCAACAGGTAT
CAAAATATTTAGCTGGATCGCAACTATGTGGGGTGGATCGATTACTTTCCCAACGCCTATGCTATTCTCTATAGGATTTATTAT
ATTATTCACGATTGGCGGCGTAACTGGCATAATCTTATCAAACTCGGCACTTGATAGAGTTCTGCACGATACATATTATGTTGT
GGCACATTTCCATTATACGATGTCACTCGGTGCTTTATTCACTGCATTTGCCGGCTTTTATTATTGGTTCGGTAAAATATCAGG
CAAGCAATATCCCGAAATCTTAGGCAAAATCCATTTCTGGATTACTTTTGTCGGTGTTAATCTAACTTTCTTTAAAAAAGCTGG
AAGATGCTGGGGCAATGGATTATACAATCATTGTTTCGGCTACTGCATCGGAAGCTGCTGCATTACAATTTGTTGCTCCTTATG
CCGCATGTAGTATGGGTGAGTATTTCCGTGATAACGGCAAGCATGCACTTATTATTTATGATGATTTAAGTAAACATGCTGTCG
CATATAGACAAATTTCATTGTTACTTAGAAGACCGCCCGGACGTGAAGCATATCCCGGTGACATATTTTACTTGCATTCAAGAT
TACTTGAGTGTGCTGCTAAAATGTCAGAGGAGAAAGGCGGCGGTTCACTTACGGCACTTCCTATAATCGAAACCCAAGCAGGTG
ACGTATCTGCTTATATTCCAACAAACGTTATTTCTATTACTGACGGTCAAATTTTCTTAGAAAGCGAGCTGTTTTATAAAGGTA
TAAGACCGGCTGTTAATGGATGGTTACTTCAAATGGTAGTGCTAATGTTTCTGGTATGGATATTGAATTAATTAATGCTCTTGC
TGCAAAAATAGGTATTAATATCGAGTACCACCAAGATAATTGGTATCAAGATCAGTTAGATATTCAAAGCGGTGCTGCTGACAT
>HJ-04-_Haemaphysalis_juxtakochi_BRA
TAACAAAAGCATTAGTGATTTTAATAAAAGATTTTCCGGTCTTTCTTTCGGTAGTCAAAAAAAGGAAGCTAGTTAATTTCTATA
ATCGGGTGGAGACATTTCCGCATAGATCAAGAACGTTTCCAGTGTGTCATCACGTTGGCGGCTTTTTTACGTGGATCGAGAGTC
GTCATTGCGAGTGAATGAAATGAGCGTGGCAATCCAGAAAATAATAAAAAATACTAATTTTATTAGTATTTTTAATTGGATCCC
CTGAATAAATCACGGGATGATAGGGGGAGAATGATCCACGTAACACACATTACTATGGGGTAAAAACTTACTCTAATCATTTAT
GTCGCTAATCTTTGTTTATAATATTAATAAACAAAGTCATCAAATTTCTTTATTGCGGGGTGGAGCAGCTCGGTAGCTATAACT
TATATAGATGGTGATCAAGGAATTTTGCGGCATCGTGGTTATGATATAAAAGATTTAGCCGAAAAAAGCGACTTTTTAGAGGTG
GCATATTTATTGATTTATGGGGAATTACCAAATAATAAGCAGTATAATGATTTTACTAAAAAGGTTGCTCATCATGCGTTAGTT
AATGAAAGATTACATTATTTATTCCAAACGTTTTGTAGCTCTTCGCATCCTATGGCTATTATGCTTGCGGCGGTTGGTTCTCTT
TCTGCATTTTATCCTGATTTGCTGAATTTTTTTAAAGAAGCGGATTATGAACTTACAGCTATTAGAATGATAGCTAAAATACCA
ACTATTGCTGCAATGGACACTCTACCCTCCTTTAAGTAGTATAAGCGGACATCCAGGAGCAGCGGTTGATATGGCTATTTTCAG
TCTGCATTTAACCGGTCTTTCATCAATACTTGGCTCAATCAACTTAATCGTTACTATCTTTAATATGAGAGCACCCGGGATGGG
ACTATTCAAAATGCCGTTATTTGTCTGGTCTATTTTAGTTACTGCATTCTTGATAATTTTAGCTATGCCAGTGCTTGGCGGAGC
TATTACTATGTTACTTACCGATCGTAATTTCGGTACTACTTTCTTTAAAACTGATGGTGGTGGTGATCCAGTATTATTTCAGCA
CTTATTTTGGTTTTTTGGTCACCCTGAAGTATATATTGTAATACTTCCAGGTTTTGGTATTGTAAGCCAAGTTATTTCCACTTT
CTCACGTAAACCAATATTTGGCTATCAAGGCATGGTTGGAGCCATGGTAATAATCGGCTTTGTCGGGTTTATTGTATGGGCTCA
CCATATGTTTACAGTTGGGCTTTCTTACAACGCACTTATATATTTTACTGCCGGAACAATGATTATCGCAATTCCAACAGGTAT
CAAAATATTTAGCTGGATCGCAACTATGTGGGGTGGATCGATTACTTTCCCAACGCCTATGCTATTCTCTATAGGATTTATTAT
ATTATTCACGATTGGCGGCGTAACTGGCATAATCTTATCAAACTCGGCACTTGATAGAGTTCTGCACGATACATATTATGTTGT
GGCACATTTCCATTATACGATGTCGCTCGGTGCTTTATTCACTGCATTTGCCGGCTTTTATTATTGGTTCGGTAAAATATCAGG
CAAGCAATATCCCGAAATCTTAGGCAAAATCCATTTCTGGATTACTTTTGTCGGTGTTAATCTAACTTTCTTTAAAAAAGCTGG

AAGATGCTGGGGCAATGGATTATACAATCATTGTTTCGGCTACTGCATCGGAAGCTGCTGCATTACAATTTGTTGCTCCTTATG
CCGCATGTAGTATGGGTGAGTATTTCCGTGATAACGGCAAGCATGCACTTATTATTTATGATGATTTAAGTAAACATGCTGTCG
CATATAGACAAATTTCATTGTTACTTAGAAGACCGCCCGGACGTGAAGCATATCCCGGTGACGTATTTTACTTGCATTCAAGAT
TACTTGAGTGTGCTGCTAAAATGTCAGAGGAGAAAGGCGGCGGTTCACTTACGGCACTTCCTATAATCGAAACCCAAGCAGGTG
ACGTATCTGCTTATATTCCAACAAACGTTATTTCTATTACTGACGGTCAAATTTTCTTAGAAAGCGAGCTGTTTTATAAAGGTA
TAAGACCGGCTGTTAATGGATGGTTACTTCAAATGGTAGTGCTAATGTTTCTGGTATGGATATTGAATTAATTAATGCTCTTGC
TGCAAAAATAGGTATTAATATCGAGTACCACCAAGATAATTGGTATCAAGATCAGTTAGATATTCAAAGCGGTGCTGCTGACAT
>IL-Mogi-_Ixodes_loricatus_BRA
TAACAAAAGCATTAGTGATTTTAATAAAAGATTTTCCGGTCTTTCTTTCGGTAGTCAAAAAAAGGAAGCTAGTTAATTTCTATA
ATCGGGTGGAGACATTTCCGCATAGATCAAGAACGTTTCCAGTGTGTCATCACGTTGGCGGCTTTTTTACGTGGATCGAGAGTC
GTCATTGCGAGTGAATGAAATGAGCGTGGCAATCCAGAAAATAATAAAAAATACTAATTTTATTAGTATTTTTAATTGGATCCC
CTGAATAAATCACGGGATGATAGGGGGAGAATGATCCACGTAACACACATTACTATGGGGTAAAAACTTACTCTAATCATTTAT
GTCGCTAATCTTTGTTTATAATATTAATAAACAAAGTCATCAAATTTCTTTATTGCGGGGTGGAGCAGCTCGGTAGCTATAACT
TATATAGATGGTGATCAAGGAATTTTGCGGCATCGTGGTTATGATATAAAAGATTTAGCCGGAAAAAGCGACTTTTTAGAGGTG
GCATATTTATTGATTTATGGGGAATTACCAAATAATAAGCAGTATAATGATTTTACTAAAAAGGTTGCTCATCATGCGTTAGTT
AATGAAAGATTACATTATTTATTCCAAACGTTTTGTAGCTCTTCGCATCCTATGGCTATTATGCTTGCGGCGGTTGGTTCTCTT
TCTGCATTTTATCCTGATTTACTGAATTTTTTTAAAGAAGCGGATTATGAACTTACAGCTATTAGAATGATAGCTAAAATACCA
ACTATTGCTGCAATGGACACTCTACCCTCCTTTAAGTAGTATAAGCGGACATCCAGGAGCAGCGGTTGATATGGCTATTTTCAG
TCTGCATTTAACCGGTCTTTCATCAATACTTGGCTCAATCAACTTAATCGTTACTATCTTTAATATGAGAGCACCCGGGATGGG
ACTATTCAAAATGCCGTTATTTGTCTGGTCTATTTTAGTTACTGCATTCTTGATAATTTTAGCTATGCCAGTGCTTGGCGGAGC
TATTACTATGTTACTTACCGATCGTAATTTCGGTACTACTTTCTTTAAAACTGATGGTGGTGGTGATCCAGTATTATTTCAGCA
CTTATTTTGGTTTTTTGGTCACCCTGAAGTATATATTGTAATACTTCCAGGTTTTGGTATTGTAAGCCAAGTTATTTCCACTTT
CTCACGTAAACCAATATTTGGCTATCAAGGCATGGTTGGAGCCATGGTAATAATCGGCTTTGTCGGGTTTATTGTATGGGCTCA
CCATATGTTTACAGTTGGGCTTTCTTACAACGCACTTATATATTTTACTGCCGGAACAATGATTATCGCAATTCCAACAGGTAT
CAAAATATTTAGCTGGATCGCAACTATGTGGGGTGGATCGATTACTTTCCCAACGCCTATGCTATTCTCTATAGGATTTATTAT
ATTATTCACGATTGGCGGCGTAACTGGCATAATCTTATCAAACTCGGCACTTGATAGAGTTCTGCACGATACATATTATGTTGT
GGCACATTTCCATTATACGATGTCGCTCGGTGCTTTATTCACTGCATTTGCCGGCTTTTATTATTGGTTCGGTAAAATATCAGG
CAAGCAATATCCCGAAATCTTAGGCAAAATCCATTTCTTGATTACTTTTGTCGGTGTTAATCTAACTTTCTTTAAAAAAGCTGG
AAGATGCTGGGGCAATGGATTATACAATCATTGTTTCGGCTACTGCATCGGAAGCTGCTGCATTACAATTTGTTGCTCCTTATG
CCGCATGTAGTATGGGTGAGTATTTCCGTGATAACGGCAAGCATGCACTTATTATTTATGATGATTTAAGTAAACATGCTGTCG
CATATAGACAAATTTCATTGTTACTTAGAAGACCGCCCGGACGTGAAGCATATCCCGGTGACGTATTTTACTTGCATTCAAGAT
TACTTGAGTGTGCTGCTAAAATGTCAGAGGAGAAAGGCGGCGGTTCACTTACGGCACTTCCTATAATCGAAACCCAAGCAGGTG
ACGTATCTGCTTATATTCCAACAAACGTTATTTCTATTACTGACGGTCAAATTTTCTTAGAAAGCGAGCTGTTTTATAAAGGTA
TAAGACCGGCTGTTAATGGATGGTTACTTCAAATGGTAGTGCTAATGTTTCTGGTATGGATATTGAATTAATTAATGCTCTTGC
TGCAAAAATAGGTATTAATATCGAGTACCACCAAGATAATTGGTATCAAGATCAGTTAGATATTCAAAGCGGTGCTGCTGACAT
>Ap_GSV_136-_Amblyomma_parvum_BRA
TAACAAAAGCATTAGTGATTTTAATAAAAGATTTTCCGGTCTTTCTTTCGGTAGTCAAAAAAAGGAAGCTAGTTAATTTCTATA
ATCGGGTGGAGACATTTCCGCATAGATCAAGAACGTTTCCAGTGTGTCATCACGTTGGCGGCTTTTTTACGTGGATCGAGAGTC
GTCATTGCGAGTGAATGAAATGAGCGTGGCAATCCAGAAAATAATAAAAAATACTAATTTTATTAGTATTTTTAATTGGATCCC
CTGAATAAATCACGGGATGATAGGGGGAGAATGATCCACGTAACACACATTACTATGGGGTAAAAACTTACTCTAATCATTTAT
GTCGCTAATCTTTGTTTATAATATTAATAAACAAAGTCATCAAATTTCTTTATTGCGGGGTGGAGCAGCTCGGTAGCTATAACT
TATATAGATGGTGATCAAGGAATTTTGCGGCATCGTGGTTATGATATAAAAGATTTAGCCGAAAAAAGCGACTTTTTAGAGGTG
GCATATTTATTGATTTATGGGGAATTACCAAATAATAAGCAGTATAATGATTTTACTAAAAAGGTTGCTCATCATGCGTTAGTT
AATGAAAGATTACATTATTTATTCCAAACGTTTTGTAGCTCTTCGCATCCTATGGCTATTATGCTTGCGGCGGTTGGTTCTCTT
TCTGCATTTTATCCTGATTTGCTGAATTTTTTTAAAGAAGCGGATTATGAACTTACAGCTATTAGAATGATAGCTAAAATACCA
ACTATTGCTGCAATGGACACTCTACCCTCCTTTAAGTAGTATAAGCGGACATCCAGGAGCAGCGGTTGATATGGCTATTTTCAG
TCTGCATTTAACCGGTCTTTCATCAATACTTGGCTCAATCAACTTAATCGTTACTATCTTTAATATGAGAGCACCCGGGATGGG
ACTATTCAAAATGCCGTTATTTGTCTGGTCTATTTTAGTTACTGCATTCTTGATAATTTTAGCTATGCCAGTGCTTGGCGGAGC
TATTACTATGTTACTTACCGATCGTAATTTCGGTACTACTTTCTTTAAAACTGATGGTGGTGGTGATCCAGTATTATTTCAGCA
CTTATTTTGGTTTTTTGGTCACCCTGAAGTATATATTGTAATACTTCCAGGTTTTGGTATTGTAAGCCAAGTTATTTCCACTTT
CTCACGTAAACCAATATTTGGCTATCAAGGCATGGTTGGAGCCATGGTAATAATCGGCTTTGTCGGGTTTATTGTATGGGCTCA
CCATATGTTTACAGTTGGGCTTTCTTACAACGCACTTATATATTTTACTGCCGGAACAATGATTATCGCAATTCCAACAGGTAT
CAAAATATTTAGCTGGATCGCAACTATGTGGGGTGGATCGATTACTTTCCCAACGCCTATGCTATTCTCTATAGGATTTATTAT
ATTATTCACGATTGGCGGCGTAACTGGCATAATCTTATCAAACTCGGCACTTGATAGAGTTCTGCACGATACATATTATGTTGT
GGCACATTTCCATTATACGATGTCGCTCGGTGCTTTATTCACTGCATTTGCCGGCTTTTATTATTGGTTCGGTAAAATATCAGG
CAAGCAATATCCCGAAATCTTAGGCAAAATCCATTTCTGGATTACTTTTGTCGGTGTTAATCTAACTTTCTTTAAAAAAGCTGG
AAGATGCTGGGGCAATGGATTATACAATCATTGTTTCGGCTACTGCATCGGAAGCTGCTGTATTACAATTTGTTGCTCCTTATG
CCGCATGTAGTATGGGTGAGTATTTCCGTGATAACGGCAAGCATGCACTTATTATTTATGATGATTTAAGTAAACATGCTGTCG
CATATAGACAAATTTCATTGTTACTTAGAAGACCGCCCGGACGTGAAGCATATCCCGGTGACGTATTTTACTTGCATTCAAGAT
TACTTGAGTGTGCTGCTAAAATGTCAGAGGAGAAAGGCGGCGGTTCACTTACGGCACTTCCTATAATCGAAACCCAAGCAGGTG
ACGTATCTGCTTATATTCCAACAAACGTTATTTCTATTACTGACGGTCAAATTTTCTTAGAAAGCGAGCTGTTTTATAAAGGTA
TAAGACCGGCTGTTAATGGATGGTTACTTCAAATGGTAGTGCTAATGTTTCTGGTATGGATATTGAATTAATTAATGCTCTTGC
TGCAAAAATAGGTATTAATATCGAGTACCACCAAGATAATTGGTATCAAGATCAGTTAGATATTCAAAGCGGTGCTGCTGACAT

>IL-RS1-_Ixodes_loricatus_BRA
TAACAAAAGCATTAGTGATTTTAATAAAAGATTTTCCGGTCTTTCTTTCGGTAGTCAAAAAAAGGAAGCTAGTTAATTTCTATA
ATCGGGTGGAGACATTTCCGCATAGATCAAGAACGTTTCCAGTGTGTCATCACGTTGGCGGCTTTTTTACGTGGATCGAGAGTC
GTCATTGCGAGTGAATGAAATGAGCGTGGCAATCCAGAAAATAATAAAAAATACTAATTTTATTAGTATTTTTAATTGGATCCC
CTGAATAAATCACGGGATGATAGGGGGAGAATGATCCACGTAACACACATTACTATGGGGTAAAAACTTACTCTAATCATTTAT
GTCGCTAATCTTTGTTTATAATATTAATAAACAAAGTCATCAAATTTCTTTATTGCGGGGTGGAGCAGCTCGGTAGCTATAACT
TATATAGATGGTGATCAAGGAATTTTGCGGCATCGTGGTTATGATATAAAAGATTTAGCCGGAAAAAGCGACTTTTTAGAGGTG
GCATATTTATTGATTTATGGGGAATTACCAAATAATAAGCAGTATAATGATTTTACTAAAAAGGTTGCTCATCATGCGTTAGTT
AATGAAAGATTACATTATTTATTCCAAACGTTTTGTAGCTCTTCGCATCCTATGGCTATTATGCTTGCGGCGGTTGGTTCTCTT
TCTGCATTTTATCCTGATTTACTGAATTTTTTTAAAGAAGCGGATTATGAACTTACAGCTATTAGAATGATAGCTAAAATACCA
ACTATTGCTGCAATGGACACTCTACCCTCCTTTAAGTAGTATAAGCGGACATCCAGGAGCAGCGGTTGATATGGCTATTTTCAG
TCTGCATTTAACCGGTCTTTCATCAATACTTGGCTCAATCAACTTAATCGTTACTATCTTTAATATGAGAGCACCCGGGATGGG
ACTATTCAAAATGCCGTTATTTGTCTGGTCTATTTTAGTTACTGCATTCTTGATAATTTTAGCTATGCCAGTGCTTGGCGGAGC
TATTACTATGTTACTTACCGATCGTAATTTCGGTACTACTTTCTTTAAAACTGATGGTGGTGGTGATCCAGTATTATTTCAGCA
CTTATTTTGGTTTTTTGGTCACCCTGAAGTATATATTGTAATACTTCCAGGTTTTGGTATTGTAAGCCAAGTTATTTCCACTTT
CTCACGTAAACCAATATTTGGCTATCAAGGCATGGTTGGAGCCATGGTAATAATCGGCTTTGTCGGGTTTATTGTATGGGCTCA
CCATATGTTTACAGTTGGGCTTTCTTACAACGCACTTATATATTTTACTGCCGGAACAATGATTATCGCAATTCCAACAGGTAT
CAAAATATTTAGCTGGATCGCAACTATGTGGGGTGGATCGATTACTTTCCCAACGCCTATGCTATTCTCTATAGGATTTATTAT
ATTATTCACGATTGGCGGCGTAACTGGCATAATCTTATCAAACTCGGCACTTGATAGAGTTCTGCACGATACATATTATGTTGT
GGCACATTTCCATTATACGATGTCGCTCGGTGCTTTATTCACTGCATTTGCCGGCTTTTATTATTGGTTCGGTAAAATATCAGG
CAAGCAATATCCCGAAATCTTAGGCAAAATCCATTTCTTGATTACTTTTGTCGGTGTTAATCTAACTTTCTTTAAAAAAGCTGG
AAGATGCTGGGGCAATGGATTATACAATCATTGTTTCGGCTACTGCATCGGAAGCTGCTGCATTACAATTTGTTGCTCCTTATG
CCGCATGTAGTATGGGTGAGTATTTCCGTGATAACGGCAAGCATGCACTTATTATTTATGATGATTTAAGTAAACATGCTGTCG
CATATAGACAAATTTCATTGTTACTTAGAAGACCGCCCGGACGTGAAGCATATCCCGGTGACGTATTTTACTTGCATTCAAGAT
TACTTGAGTGTGCTGCTAAAATGTCAGAGGAGAAAGGCGGCGGTTCACTTACGGCACTTCCTATAATCGAAACCCAAGCAGGTG
ACGTATCTGCTTATATTCCAACAAACGTTATTTCTATTACTGACGGTCAAATTTTCTTAGAAAGCGAGCTGTTTTATAAAGGTA
TAAGACCGGCTGTTAATGGATGGTTACTTCAAATGGTAGTGCTAATGTTTCTGGTATGGATATTGAATTAATTAATGCTCTTGC
TGCAAAAATAGGTATTAATATCGAGTACCACCAAGATAATTGGTATCAAGATCAGTTAGATATTCAAAGCGGTGCTGCTGACAT
>RB-CL-_Ixodes_loricatus_BRA
TAACAAAAGCATTAGTGATTTTAATAAAAGATTTTCCGGTCTTTCTTTCGGTAGTCAAAAAAAGGAAGCTAGTTAATTTCTATA
ATCGGGTGGAGACATTTCCGCATAGATCAAGAACGTTTCCAGTGTGTCATCACGTTGGCGGCTTTTTTACGTGGATCGAGAGTC
GTCATTGCGAGTGAATGAAATGAGCGTGGCAATCCAGAAAATAATAAAAAATACTAATTTTATTAGTATTTTTAATTGGATCCC
CTGAATAAATCACGGGATGATAGGGGGAGAATGATCCACGTAACACACATTACTATGGGGTAAAAACTTACTCTAATCATTTAT
GTCGCTAATCTTTGTTTATAATATTAATAAACAAAGTCATCAAATTTCTTTATTGCGGGGTGGAGCAGCTCGGTAGCTATAACT
TATATAGATGGTGATCAAGGAATTTTGCGGCATCGTGGTTATGATATAAAAGATTTAGCCGGAAAAAGCGACTTTTTAGAGGTG
GCATATTTATTGATTTATGGGGAATTACCAAATAATAAGCAGTATAATGATTTTACTAAAAAGGTTGCTCATCATGCGTTAGTT
AATGAAAGATTACATTATTTATTCCAAACGTTTTGTAGCTCTTCGCATCCTATGGCTATTATGCTTGCGGCGGTTGGTTCTCTT
TCTGCATTTTATCCTGATTTACTGAATTTTTTTAAAGAAGCGGATTATGAACTTACAGCTATTAGAATGATAGCTAAAATACCA
ACTATTGCTGCAATGGACACTCTACCCTCCTTTAAGTAGTATAAGCGGACATCCAGGAGCAGCGGTTGATATGGCTATTTTCAG
TCTGCATTTAACCGGTCTTTCATCAATACTTGGCTCAATCAACTTAATCGTTACTATCTTTAATATGAGAGCACCCGGGATGGG
ACTATTCAAAATGCCGTTATTTGTCTGGTCTATTTTAGTTACTGCATTCTTGATAATTTTAGCTATGCCAGTGCTTGGCGGAGC
TATTACTATGTTACTTACCGATCGTAATTTCGGTACTACTTTCTTTAAAACTGATGGTGGTGGTGATCCAGTATTATTTCAGCA
CTTATTTTGGTTTTTTGGTCACCCTGAAGTATATATTGTAATACTTCCAGGTTTTGGTATTGTAAGCCAAGTTATTTCCACTTT
CTCACGTAAACCAATATTTGGCTATCAAGGCATGGTTGGAGCCATGGTAATAATCGGCTTTGTCGGGTTTATTGTATGGGCTCA
CCATATGTTTACAGTTGGGCTTTCTTACAACGCACTTATATATTTTACTGCCGGAACAATGATTATCGCAATTCCAACAGGTAT
CAAAATATTTAGCTGGATCGCAACTATGTGGGGTGGATCGATTACTTTCCCAACGCCTATGCTATTCTCTATAGGATTTATTAT
ATTATTCACGATTGGCGGCGTAACTGGCATAATCTTATCAAACTCGGCACTTGATAGAGTTCTGCACGATACATATTATGTTGT
GGCACATTTCCATTATACGATGTCGCTCGGTGCTTTATTCACTGCATTTGCCGGCTTTTATTATTGGTTCGGTAAAATATCAGG
CAAGCAATATCCCGAAATCTTAGGCAAAATCCATTTCTTGATTACTTTTGTCGGTGTTAATCTAACTTTCTTTAAAAAAGCTGG
AAGATGCTGGGGCAATGGATTATACAATCATTGTTTCGGCTACTGCATCGGAAGCTGCTGCATTACAATTTGTTGCTCCTTATG
CCGCATGTAGTATGGGTGAGTATTTCCGTGATAACGGCAAGCATGCACTTATTATTTATGATGATTTAAGTAAACATGCTGTCG
CATATAGACAAATTTCATTGTTACTTAGAAGACCGCCCGGACGTGAAGCATATCCCGGTGACGTATTTTACTTGCATTCAAGAT
TACTTGAGTGTGCTGCTAAAATGTCAGAGGAGAAAGGCGGCGGTTCACTTACGGCACTTCCTATAATCGAAACCCAAGCAGGTG
ACGTATCTGCTTATATTCCAACAAACGTTATTTCTATTACTGACGGTCAAATTTTCTTAGAAAGCGAGCTGTTTTATAAAGGTA
TAAGACCGGCTGTTAATGGATGGTTACTTCAAATGGTAGTGCTAATGTTTCTGGTATGGATATTGAATTAATTAATGCTCTTGC
TGCAAAAATAGGTATTAATATCGAGTACCACCAAGATAATTGGTATCAAGATCAGTTAGATATTCAAAGCGGTGCTGCTGACAT
>HJ-1-_Haemaphysalis_juxtakochi_BRA
TAACAAAAGCATTAGTGATTTTAATAAAAGATTTTCCGGTCTTTCTTTCGGTAGTCAAAAAAAGGAAGCTAGTTAATTTCTATA
ATCGGGTGGAGACATTTCCGCATAGATCAAGAACGTTTCCAGTGTGTCATCACGTTGGCGGCTTTTTTACGTGGATCGAGAGTC
GTCATTGCGAGTGAATGAAATGAGCGTGGCAATCCAGAAAATAATAAAAAATACTAATTTTATTAGTATTTTTAATTGGATCCC
CTGAATAAATCACGGGATGATAGGGGGAGAATGATCCACGTAACACACATTACTATGGGGTAAAAACTTACTCTAATCATTTAT
GTCGCTAATCTTTGTTTATAATATTAATAAACAAAGTCATCAAATTTCTTTATTGCGGGGTGGAGCAGCTCGGTAGCTATAACT
TATATAGATGGTGATCAAGGAATTTTGCGGCATCGTGGTTATGATATAAAAGATTTAGCCGAAAAAAGCGACTTTTTAGAGGTG

GCATATTTATTGATTTATGGGGAATTACCAAATAATAAGCAGTATAATGATTTTACTAAAAAGGTTGCTCATCATGCGTTAGTT
AATGAAAGATTACATTATTTATTCCAAACGTTTTGTAGCTCTTCGCATCCTATGGCTATTATGCTTGCGGCGGTTGGTTCTCTT
TCTGCATTTTATCCTGATTTGCTGAATTTTTTTAAAGAAGCGGATTATGAACTTACAGCTATTAGAATGATAGCTAAAATACCA
ACTATTGCTGCAATGGACACTCTACCCTCCTTTAAGTAGTATAAGCGGACATCCAGGAGCAGCGGTTGATATGGCTATTTTCAG
TCTGCATTTAACCGGTCTTTCATCAATACTTGGCTCAATCAACTTAATCGTTACTATCTTTAATATGAGAGCACCCGGGATGGG
ACTATTCAAAATGCCGTTATTTGTCTGGTCTATTTTAGTTACTGCATTCTTGATAATTTTAGCTATGCCAGTGCTTGGCGGAGC
TATTACTATGTTACTTACCGATCGTAATTTCGGTACTACTTTCTTTAAAACTGATGGTGGTGGTGATCCAGTATTATTTCAGCA
CTTATTTTGGTTTTTTGGTCACCCTGAAGTATATATTGTAATACTTCCAGGTTTTGGTATTGTAAGCCAAGTTATTTCCACTTT
CTCACGTAAACCAATATTTGGCTATCAAGGCATGGTTGGAGCCATGGTAATAATCGGCTTTGTCGGGTTTATTGTATGGGCTCA
CCATATGTTTACAGTTGGGCTTTCTTACAACGCACTTATATATTTTACTGCCGGAACAATGATTATCGCAATTCCAACAGGTAT
CAAAATATTTAGCTGGATCGCAACTATGTGGGGTGGATCGATTACTTTCCCAACGCCTATGCTATTCTCTATAGGATTTATTAT
ATTATTCACGATTGGCGGCGTAACTGGCATAATCTTATCAAACTCGGCACTTGATAGAGTTCTGCACGATACATATTATGTTGT
GGCACATTTCCATTATACGATGTCGCTCGGTGCTTTATTCACTGCATTTGCCGGCTTTTATTATTGGTTCGGTAAAATATCAGG
CAAGCAATATCCCGAAATCTTAGGCAAAATCCATTTCTGGATTACTTTTGTCGGTGTTAATCTAACTTTCTTTAAAAAAGCTGG
AAGATGCTGGGGCAATGGATTATACAATCATTGTTTCGGCTACTGCATCGGAAGCTGCTGCATTACAATTTGTTGCTCCTTATG
CCGCATGTAGTATGGGTGAGTATTTCCGTGATAACGGCAAGCATGCACTTATTATTTATGATGATTTAAGTAAACATGCTGTCG
CATATAGACAAATTTCATTGTTACTTAGAAGACCGCCCGGACGTGAAGCATATCCCGGTGACGTATTTTACTTGCATTCAAGAT
TACTTGAGTGTGCTGCTAAAATGTCAGAGGAGAAAGGCGGCGGTTCACTTACGGCACTTCCTATAATCGAAACCCAAGCAGGTG
ACGTATCTGCTTATATTCCAACAAACGTTATTTCTATTACTGACGGTCAAATTTTCTTAGAAAGCGAGCTGTTTTATAAAGGTA
TAAGACCGGCTGTTAATGGATGGTTACTTCAAATGGTAGTGCTAATGTTTCTGGTATGGATATTGAATTAATTAATGCTCTTGC
TGCAAAAATAGGTATTAATATCGAGTACCACCAAGATAATTGGTATCAAGATCAGTTAGATATTCAAAGCGGTGCTGCTGACAT
>Rb_MS_PSEUDO-_Amblyomma_pseudoconcolor_BRA
TAACAAAAGCATTAGTGATTTTAATAAAAGATTTTCCGGTCTTTCTTTCGGTAGTCAAAAAAAGGAAGCTAGTTAATTTCTATA
ATCGGGTGGAGACATTTCCGCATAGATCAAGAACGTTTCCAGTGTGTCATCACGTTGGCGGCTTTTTTACGTGGATCGAGAGTC
GTCATTGCGAGTGAATGAAATGAGCGTGGCAATCCAGAAAATAATAAAAAATACTAATTTTATTAGTATTTTTAATTGGATCCC
CTGAATAAATCACGAGATGATAGGGGGAGAATGATCCACGTAACACACATTACTATGGGGTAAAACTTACTCTAATCATTGAT
GTCGCTAATCTTTGTTTATAATATTAATAAACAAAGTCATCAAATTTCTTTATTGCGGGGTGGAGCAGCTCGGTAGCTATAACT
TATATAGATGGTGATCAAGGAATTTTGCGGCATCGTGGTTATGATATAAAAGATTTAGCCGAAAAAAGCGACTTTTTAGAGGTG
GCATATTTATTGATTTATGGGGAATTACCAAATAATAAGCAGTATAATGATTTTACTAAAAAGGTTGCTCATCATGCGTTAGTT
AATGAAAGATTACATTATTTATTCCAAACGTTTTGTAGCTCTTCGCATCCTATGGCTATTATGCTTGCGGCGGTTGGTTCTCTT
TCTGCATTTTATCCTGATTTGCTGAATTTTTTTAAAGAAGCGGATTATGAACTTACAGCTATTAGAATGATAGCTAAAATACCA
ACTATTGCTGCAATGGACACTCTACCCTCCTTTAAGTAGTATAAGCGGACATCCAGGAGCAGCGGTTGATATGGCTATTTTCAG
TCTGCATTTAACCGGTCTTTCATCAATACTTGGCTCAATCAACTTAATCGTTACTATCTTTAATATGAGAGCACCCGGGATGGG
ACTATTCAAAATGCCGTTATTTGTCTGGTCTATTTTAGTTACTGCATTCTTGATAATTTTAGCTATGCCAGTGCTTGGCGGAGC
TATTACTATGTTACTTACCGATCGTAATTTCGGTACTACTTTCTTTAAAACTGATGGTGGTGGTGATCCAGTATTATTTCAGCA
CTTATTTTGGTTTTTTGGTCACCCTGAAGTATATATTGTAATACTTCCAGGTTTTGGTATTGTAAGCCAAGTTATTTCCACTTT
CTCACGTAAACCAATATTTGGCTATCAAGGCATGGTTGGAGCCATGGTAATAATCGGCTTTGTCGGGTTTATTGTATGGGCTCA
CCATATGTTTACAGTTGGGCTTTCTTACAACGCACTTATATATTTTACTGCCGGAACAATGATTATCGCAATTCCAACAGGTAT
CAAAATATTTAGCTGGATCGCAACTATGTGGGGTGGATCGATTACTTTCCCAACGCCTATGCTATTCTCTATAGGATTTATTAT
ATTATTCACGATTGGCGGCGTAACTGGCATAATCTTATCAAACTCGGCACTTGATAGAGTTCTGCACGATACATATTATGTTGT
GGCACATTTCCATTATACGATGTCGCTCGGTGCTTTATTCACTGCATTTGCCGGCTTTTATTATTGGTTCGGTAAAATATCAGG
CAAGCAATATCCCGAAATCTTAGGCAAAATCCATTTCTGGATTACTTTTGTCGGTGTTAATCTAACTTTCTTTAAAAAAGCTGG
AAGATGCTGGGGCAATGGATTATACAATCATTGTTTCGGCTACTGCATCGGAAGCTGCTGCATTACAATTTGTTGCTCCTTATG
CCGCATGTAGTATGGGTGAGTATTTCCGTGATAACGGCAAGCATGCACTTATTATTTATGATGATTTAAGTAAACATGCTGTCG
CATATAGACAAATTTCATTGTTACTTAGAAGACCGCCCGGACGTGAAGCATATCCCGGTGACGTATTTTACTTGCATTCAAGAT
TACTTGAGTGTGCTGCTAAAATGTCAGAGGAGAAAGGCGGCGGTTCACTTACGGCACTTCCTATAATCGAAACCCAAGCAGGTG
ACGTATCTGCTTATATTCCAACAAACGTTATTTCTATTACTGACGGTCAAATTTTCTTAGAAAGCGAGCTGTTTTATAAAGGTA
TAAGACCGGCTGTTAATGGATGGTTACTTCAAATGGTAGTGCTAATGTTTCTGGTATGGATATTGAATTAATTAATGCTCTTGC
TGCAAAAATAGGTATTAATATCGAGTACCACCAAGATAATTGGTATCAAGATCAGTTAGATATTCAAAGCGGTGCTGCTGACAT
>A.ovale_51-_Amblyomma_ovale_BRA
TAACAAAAGCATTAGTGATTTTAATAAAAGATTTTCCGGTCTTTCTTTCGGTAGTCAAAAAAAGGAAGCTAGTTAATTTCTATA
ATCGGGTGGAGACATTTCCGCATAGATCAAGAACGTTTCCAGTGTGTCATCACGTTGGCGGCTTTTTTACGTGGATCGAGAGTC
GTCATTGCGAGTGAATGAAATGAGCGTGGCAATCCAGAAAATAATAAAAAATACTAATTTTATTAGTATTTTTAATTGGATCCC
TTGAATAAATCACGGGATGATAGGGGGAGAATGATCCACGTAACACACATTACTATGGGGTAAAACTTACTCTAATCATTTAT
GTCGCTAATCTTTGTTTATAATATTAATAAACAAAGTCATCAAATTTCTTTATTGCGGGGTGGAGCAGCTCGGTAGCTATAACT
TATATAGATGGTGATCAAGGAATTTTGCGGCATCGTGGTTATGATATAAAAGATTTAGCCGAAAAAAGCGACTTTTTAGAGGTG
GCATATTTATTGATTTATGGGGAATTACCAAATAATAAGCAGTATAATGATTTTACTAAAAAGGTTGCTCATCATGCGTTAGTT
AATGAAAGATTACATTATTTATTCCAAACGTTTTGTAGCTCTTCGCATCCTATGGCTATTATGCTTGCGGCGGTTGGTTCTCTT
TCTGCATTTTATCCTGATTTGCTGAATTTTTTTAAAGAAGCGGATTATGAACTTACAGCTATTAGAATGATAGCTAAAATACCA
ACTATTGCTGCAATGGACACTCTACCCTCCTTTAAGTAGTATAAGCGGACATCCAGGAGCAGCGGTTGATATGGCTATTTTCAG
TCTGCATTTAACCGGTCTTTCATCAATACTTGGCTCAATCAACTTAATCGTTACTATCTTTAATATGAGAGCACCCGGGATGGG
ACTATTCAAAATGCCGTTATTTGTCTGGTCTATTTTAGTTACTGCATTCTTGATAATTTTAGCTATGCCAGTGCTTGGCGGAGC
TATTACTATGTTACTTACCGATCGTAATTTCGGTACTACTTTCTTTAAAACTGATGGTGGTGGTGATCCAGTATTATTTCAGCA

CTTATTTTGGTTTTTTGGTCACCCTGAAGTATATATTGTAATACTTCCAGGTTTTGGTATTGTAAGCCAAGTTATTTCCACTTT
CTCACGTAAACCAATATTTGGCTATCAAGGCATGGTTGGAGCCATGGTAATAATCGGCTTTGTCGGGTTTATTGTATGGGCTCA
CCATATGTTTACAGTTGGGCTTTCTTACAACGCACTTATATATTTTACTGCCGGAACAATGATTATCGCAATTCCAACAGGTAT
CAAAATATTTAGCTGGATCGCAACTATGTGGGGTGGATCGATTACTTTCCCAACGCCTATGCTATTCTCTATAGGATTTATTAT
ATTATTCACGATTGGCGGCGTAACTGGCATAATCTTATCAAACTCGGCACTTGATAGAGTTCTGCACGATACATATTATGTTGT
GGCACATTTCCATTATACGATGTCACTCGGTGCTTTATTCACTGCATTTGCCGGCTTTTATTATTGGTTCGGTAAAATATCAGG
CAAGCAATATCCCGAAATCTTAGGCAAAATCCATTTCTGGATTACTTTTGTCGGTGTTAATCTAACTTTCTTTAAAAAAGCTGG
AAGATGCTGGGGCAATGGATTATACAATCATTGTTTCGGCTACTGCATCGGAAGCTGCTGCATTACAATTTGTTGCTCCTTATG
CCGCATGTAGTATGGGTGAGTATTTCCGTGATAACGGCAAGCATGCACTTATTATTTATGATGATTTAAGTAAACATGCTGTCG
CATATAGACAAATTTCATTGTTACTTAGAAGACCGCCCGGACGTGAAGCATATCCCGGTGACATATTTTACTTGCATTCAAGAT
TACTTGAGTGTGCTGCTAAAATGTCAGAGGAGAAAGGCGGCGGTTCACTTACGGCACTTCCTATAATCGAAACCCAAGCAGGTG
ACGTATCTGCTTATATTCCAACAAACGTTATTTCTATTACTGACGGTCAAATTTTCTTAGAAAGCGAGCTGTTTTATAAAGGTA
TAAGACCGGCTGTTAATGGATGGTTACTTCAAATGGTAGTGCTAATGTTTCTGGTATGGATATTGAATTAATTAATGCTCTTGC
TGCAAAAATAGGTATTAATATCGAGTACCACCAAGATAATTGGTATCAAGATCAGTTAGATATTCAAAGCGGTGCTGCTGACAT
>An4-_Amblyomma_neumanni_ARG
TAACAAAAGCATTAGTGATTTTAATAAAAGATTTTCCGGTCTTTCTTTCGGTAGTCAAAAAAAGGAAGCTAGTTAATTTCTATA
ATCGGGTGGAGACATTTCCGCATAGATCAAGAACGTTTCCAGTGTGTCATCACGTTGGCGGCTTTTTTACGTGGATCGAGAGTC
GTCATTGCGAGTGAATGAAATGAGCGTGGCAATCCAGAAAATAATAAAAAATACTAATTTTATTAGTATTTTTAATTGGATCCC
CTGAATAAATCACGGGATGATAGGGGGAGAATGATCCACGTAACACACATTACTATGGGGTAAAAACTTACTCTAATCATTTAT
GTCGCTAATCTTTGTTTATAATATTAATAAACAAAGTCATCAAATTTCTTTATTGCGGGGTGGAGCAGCTCGGTAGCTATAACT
TATATAGATGGTGATCAAGGAATTTTGCGGCATCGTGGTTATGATATAAAAGATTTAGCCGAAAAAAGCGACTTTTTAGAGGTG
GCATATTTATTGATTTATGGGGAATTACCAAATAATAAGCAGTATAATGATTTTACTAAAAAGGTTGCTCATCATGCGTTAGTT
AATGAAAGATTACATTATTTATTCCAAACGTTTTGTAGCTCTTCGCATCCTATGGCTATTATGCTTGCGGCGGTTGGTTCTCTT
TCTGCATTTTATCCTGATTTGCTGAATTTTTTTAAAGAAGCGGATTATGAACTTACAGCTATTAGAATGATAGCTAAAATACCA
ACTATTGCTGCAATGGACACTCTACCCTCCTTTAAGTAGTATAAGCGGACATCCAGGAGCAGCGGTTGATATGGCTATTTTCAG
TCTGCATTTAACCGGTCTTTCATCAATACTTGGCTCAATCAACTTAATCGTTACTATCTTTAATATGAGAGCACCCGGGATGGG
ACTATTCAAAATGCCGTTATTTGTCTGGTCTATTTTAGTTACTGCATTCTTGATAATTTTAGCTATGCCAGTGCTTGGCGGAGC
TATTACTATGTTACTTACCGATCGTAATTTCGGTACTACTTTCTTTAAAACTGATGGTGGTGGTGATCCAGTATTATTTCAGCA
CTTATTTTGGTTTTTTGGTCACCCTGAAGTATATATTGTAATACTTCCAGGTTTTGGTATTGTAAGCCAAGTTATTTCCACTTT
CTCACGTAAACCAATATTTGGCTATCAAGGCATGGTTGGAGCCATTGTAATAATCGGCTTTGTCGGGTTTATTGTATGGGCTCA
CCATATGTTTACAGTTGGGCTTTCTTACAACGCACTTATATATTTTACTGCCGGAACAATGATTATCGCAATTCCAACAGGTAT
CAAAATATTTAGCTGGATCGCAACTATGTGGGGTGGATCGATTACTTTCCCAACGCCTATGCTATTCTCTATAGGATTTATTAT
ATTATTCACGATTGGCGGCGTAACTGGCATAATCTTATCAAACTCGGCACTTGATAGAGTTCTGCACGATACATATTATGTTGT
GGCACATTTCCATTATACGATGTCGCTCGGTGCTTTATTCACTGCATTTGCCGGCTTTTATTATTGGTTCGGTAAAATATCAGG
CAAGCAATATCCCGAAATCTTAGGCAAAATCCATTTCTGGATTACTTTTGTCGGTGTTAATCTAACTTTCTTTAAAAAAGCTGG
AAGATGCTGGGGCAATGGATTATACAATCATTGTTTCGGCTACTGCATCGGAAGCTGCTGCATTACAATTTGTTGCTCCTTATG
CCGCATGTAGTATGGGTGAGTATTTCCGTGATAACGGCAAGCATGCACTTATTATTTATGATGATTTAAGTAAACATGCTGTCG
CATATAGACAAATTTCATTGTTACTTAGAAGACCGCCCGGACGTGAAGCATATCCCGGTGACGTATTTTACTTGCATTCAAGAT
TACTTGAGTGTGCTGCTAAAATGTCAGAGGAGAAAGGCGGCGGTTCACTTACGGCACTTCCTATAATCGAAACCCAAGCAGGTG
ACGTATCTGCTTATATTCCAACAAACGTTATTTCTATTACTGACGGTCAAATTTTCTTAGAAAGCGAGCTGTTTTATAAAGGTA
TAAGACCGGCTGTTAATGGATGGTTACTTCAAATGGTAGTGCTAATGTTTCTGGTATGGATATTGAATTAATTAATGCTCTTGC
TGCAAAAATAGGTATTAATATCGAGTACCACCAAGATAATTGGTATCAAGATCAGTTAGATATTCAAAGCGGTGCTGCTGACAT
>369C-_Dermacentor_andersonii_USA
TAACAAAAGCATTAGTGATTTTAATAAAAGATTTTCCGGTCTTTCTTTCGGTAGTCAAAAGAAGGAAGCTAGTTAATTTCTATA
ATCGGGTGGAGACATTTCCGCATAGATCAAGAACGTTTCCAGTGTGTCATCACGTTGGCGGCTTTTTTACGTGGATCGAGAGTC
GTCATTGCGAGTGAATGAAATGAGCGTGGCAATCCAGAAAATAATTAAAAATACTAATTTTATTAGTATTTTTAATTGGATCCC
CTGAATAAATCACGGGATGATAGGGGGAGAATGATCCACGTAACACACATTACTATGGGGTAAAAACTTACTCTAATCATTTAT
GTCGCTAATCTTTGTTTATAATATTAATAAACAAAGTCATCAAATTTCTTTATTGCGGGGTGGAGCAGCTCGGTAGCTATAACT
TATATAGATGGTGATCAAGGAATTTTGCGGCATCGTGGTTATGATATAAAAGATTTAGCCGAAAAAAGCGACTTTTTAGAGGTG
GCATATTTATTGATTTATGGGGAATTACCAAATAATAAGCAGTATAATGATTTTACTAAAAAGGTTGCTCATCATGCGTTAGTT
AATGAAAGATTACATTATTTATTCCAAACGTTTTGTAGCTCTTCGCATCCTATGGCTATTATGCTTGCGGCGGTTGGTTCTCTT
TCTGCATTTTATCCTGATTTGCTGAATTTTTTTAAAGAAGCGGATTATGAACTTACAGCTATTAGAATGATAGCTAAAATACCA
ACTATTGCTGCAATGGACACTCTACCCTCCTTTAAGTAGTATAAGCGGACATCCAGGAGCAGCGGTTGATATGGCTATTTTCAG
TCTGCATTTAACCGGTCTTTCATCAATACTTGGCTCAATCAACTTAATCGTTACTATCTTTAATATGAGAGCACCCGGGATGGG
ACTATTCAAAATGCCGTTATTTGTCTGGTCTATTTTAGTTACTGCATTCTTGATAATTTTAGCTATGCCAGTGCTTGGCGGAGC
TATTACTATGTTACTTACCGATCGTAATTTCGGTACTACTTTCTTTAAAACTGATGGTGGTGGTGATCCAGTATTATTTCAGCA
CTTATTTTGGTTTTTTGGTCACCCTGAAGTATATATTGTAATACTTCCAGGTTTTGGTATTGTAAGCCAAGTTATTTCCACTTT
CTCACGTAAACCAATATTTGGCTATCAAGGCATGGTTGGAGCCATGGTAATAATCGGCTTTGTCGGGTTTATTGTATGGGCTCA
CCATATGTTTACAGTTGGGCTTTCTTACAACGCACTTATATATTTTACTGCCGGAACAATGATTATCGCAATTCCAACAGGTAT
CAAAATATTTAGCTGGATCGCAACTATGTGGGGTGGATCGATTACTTTCCCAACGCCTATGCTATTCTCTATAGGATTTATTAT
ATTATTCACGATTGGCGGCGTAACTGGCATAATCTTATCAAACTCGGCACTTGATAGAGTTCTGCACGATACATATTATGTTGT
GGCACATTTCCATTATACGATGTCGCTCGGTGCTTTATTCACTGCATTTGCCGGCTTTTATTATTGGTTCGGTAAAATATCAGG
CAAGCAATATCCCGAAATCTTAGGCAAAATCCATTTCTGGATTACTTTTGTCGGTGTTAATCTAACTTTCTTTAAAAAAGCTGG

AAGATGCTGGGGCAATGGATTATACAATCATTGTTTCGGCTACTGCATCGGAAGCTGCTGTATTACAATTTGTTGCTCCTTATG
CCGCATGTAGTATGGGTGAGTATTTCCGTGATAACGGCAAGCATGCACTTATTATTTATGATGATTTAAGTAAACATGCTGTCG
CATATAGACAAATTTCATTGTTACTTAGAAGACCGCCCGGACGTGAAGCATATCCCGGTGACGTATTTTACTTGCATTCAAGAT
TACTTGAGTGTGCTGCTAAAATGTCAGAGGAGAAAGGCGGCGGTTCACTTACGGCACTTCCTATAATCGAAACCCAAGCAGGTG
ACGTATCTGCTTATATTCCAACAAACGTTATTTCTATTACTGACGGTCAAATTTTCTTAGAAAGCGAGCTGTTTTATAAAGGTA
TAAGACCGGCTGTTAATGGATGGTTACTTCAAATGGTAGTGCTAATGTTTCTGGTATGGATATTGAATTAATTAATGCTCTTGC
TGCAAAAATAGGTATTAATATCGAGTACCACCAAGATAATTGGTATCAAGATCAGTTAGATATTCAAAGCGGTGCTGCTGACAT
>CA13-1-_Dermacentor_variabilis_USA
TAACAAAAGCATTAGTGATTTTAATAAAAGATTTTCCGGTCTTTCTTTCGGTAGTCAAAAAAAGGAAGCTAGTTAATTTCTATA
ATCGGGTGGAGACATTTCCGCATAGATCAAGAACGTTTCCAGTGTGTCATCACGTTGGCGGCTTTTTTACGTGGATCGAGAGTC
GTCATTGCGAGTGAATGAAATGAGCGTGGCAATCCAGAAAATAATTAAAAATACTAATAAAATTAGTATTTTTAATTGGATCCC
CTGAATAAATCACGGGATGATAGGGGGAGAATGATCCACGTAACACACATTACTATGGGGTAAAAACTTACTCTAATCATTTAT
GTCGCTAATCTTTGTTTATAATATTAATAAACAAAGTCATCAAATTTCTTTATTGCGGGGTGGAGCAGCTCGGTAGCTATAACT
TATATAGATGGTGATCAAGGAATTTTGCGGCATCGTGGTTATGATATAAAAGATTTAGCCGAAAAAAGCGACTTTTTAGAGGTG
GCATATTTATTGATTTATGGGGAATTACCAAATAATAAGCAGTATAATGATTTTACTAAAAAGGTTGCTCATCATGCGTTAGTT
AATGAAAGATTACATTATTTATTCCAAACGTTTTGTAGCTCTTCGCATCCTATGGCTATTATGCTTGCGGCGGTTGGTTCTCTT
TCTGCATTTTATCCTGATTTGCTGAATTTTTTTAAAGAAGCGGATTATGAACTTACAGCTATTAGAATGATAGCTAAAATACCA
ACTATTGCTGCAATGGACACTCTACCCTCCTTTAAGTAGTATAAGCGGACATCCAGGAGCAGCGGTTGATATGGCTATTTTCAG
TCTGCATTTAACCGGTCTTTCATCAATACTTGGCTCAATCAACTTAATCGTTACTATCTTTAATATGAGAGCACCCGGGATGGG
ACTATTCAAAATGCCGTTATTTGTCTGGTCTATTTTAGTTACTGCATTCTTGATAATTTTAGCTATGCCAGTGCTTGGCGGAGC
TATTACTATGTTACTTACAGATCGTAATTTCGGTACTACTTTCTTTAAAACTGATGGTGGTGGTGATCCAGTATTATTTCAGCA
CTTATTTTGGTTTTTTGGTCACCCTGAAGTATATATTGTAATACTTCCAGGTTTTGGTATTGTAAGCCAAGTTATTTCCACTTT
CTCACGTAAACCAATATTTGGCTATCAAGGCATGGTTGGAGCCATGGTAATAATCGGCTTTGTCGGGTTTATTGTATGGGCTCA
CCATATGTTTACAGTTGGGCTTTCTTACAACGCACTTATATATTTTACTGCCGGAACAATGATTATCGCAATTCCAACAGGTAT
CAAAATATTTAGCTGGATCGCAACTATGTGGGGTGGATCGATTACTTTCCCAACGCCTATGCTATTCTCTATAGGATTTATTAT
ATTATTCACGATTGGCGGCGTAACTGGCATAATCTTATCAAACTCGGCACTTGATAGAGTTCTGCACGATACATATTATGTTGT
GGCACATTTCCATTATACGATGTCGCTCGGTGCTTTATTCACTGCATTTGCCGGCTTTTATTATTGGTTCGGTAAAATATCAGG
CAAGCAATATCCCGAAATCTTAGGCAAAATCCATTTCTGGATTACTTTTGTCGGTGTTAATCTAACTTTCTTTAAAAAAGCTGG
AAGATGCTGGGGCAATGGATTATACAATCATTGTTTCGGCTACTGCATCGGAAGCTGCTGCATTACAATTTGTTGCTCCTTATG
CCGCATGTAGTATGGGTGAGTATTTCCGTGATAACGGCAAGCATGCACTTATTATTTATGATGATTTAAGTAAACATGCTGTCG
CATATAGACAAATTTCATTGTTACTTAGAAGACCGCCCGGACGTGAAGCATATCCCGGTGACGTATTTTACTTGCATTCAAGAT
TACTTGAGTGTGCTGCTAAAATGTCAGAGGAGAAAGGCGGCGGTTCACTTACGGCACTTCCTATAATCGAAACCCAAGCAGGTG
ACGTATCTGCTTATATTCCAACAAACGTTATTTCTATTACTGACGGTCAAATTTTCTTAGAAAGCGAGCTGTTTTATAAAGGTA
TAAGACCGGCTGTTAATGGATGGTTACTTCAAATGGTAGTGCTAATGTTTCTGGTATGGATATTGAATTAATTAATGCTCTTGC
TGCAAAAATAGGTATTAATATCGAGTACCACCAAGATAATTGGTATCAAGATCAGTTAGATATTCAAAGCGGTGTTGCTGACAT
>CA13-9-_Dermacentor_variabilis_USA
TAACAAAAGCATTAGTGATTTTAATAAAAGATTTTCCGGTCTTTCTTTCGGTAGTCAAAAAAAGGAAGCTAGTTAATTTCTATA
ATCGGGTGGAGACATTTCCGCATAGATCAAGAACGTTTCCAGTGTGTCATCACGTTGGCGGCTTTTTTACGTGGATCGAGAGTC
GTCATTGCGAGTGAATGAAATGAGCGTGGCAATCCAGAAAATAATTAAAAATACTAATAAAATTAGTATTTTTAATTGGATCCC
CTGAATAAATCACGGGATGATAGGGGGAGAATGATCCACGTAACACACATTACTATGGGGTAAAAACTTACTCTAATCATTTAT
GTCGCTAATCTTTGTTTATAATATTAATAAACAAAGTCATCAAATTTCTTTATTGCGGGGTGGAGCAGCTCGGTAGCTATAACT
TATATAGATGGTGATCAAGGAATTTTGCGGCATCGTGGTTATGATATAAAAGATTTAGCCGAAAAAAGCGACTTTTTAGAGGTG
GCATATTTATTGATTTATGGGGAATTACCAAATAATAAGCAGTATAATGATTTTACTAAAAAGGTTGCTCATCATGCGTTAGTT
AATGAAAGATTACATTATTTATTCCAAACGTTTTGTAGCTCTTCGCATCCTATGGCTATTATGCTTGCGGCGGTTGGTTCTCTT
TCTGCATTTTATCCTGATTTGCTGAATTTTTTTAAAGAAGCGGATTATGAACTTACAGCTATTAGAATGATAGCTAAAATACCA
ACTATTGCTGCAATGGACACTCTACCCTCCTTTAAGTAGTATAAGCGGACATCCAGGAGCAGCGGTTGATATGGCTATTTTCAG
TCTGCATTTAACCGGTCTTTCATCAATACTTGGCTCAATCAACTTAATCGTTACTATCTTTAATATGAGAGCACCCGGGATGGG
ACTATTCAAAATGCCGTTATTTGTCTGGTCTATTTTAGTTACTGCATTCTTGATAATTTTAGCTATGCCAGTGCTTGGCGGAGC
TATTACTATGTTACTTACAGATCGTAATTTCGGTACTACTTTCTTTAAAACTGATGGTGGTGGTGATCCAGTATTATTTCAGCA
CTTATTTTGGTTTTTTGGTCACCCTGAAGTATATATTGTAATACTTCCAGGTTTTGGTATTGTAAGCCAAGTTATTTCCACTTT
CTCACGTAAACCAATATTTGGCTATCAAGGCATGGTTGGAGCCATGGTAATAATCGGCTTTGTCGGGTTTATTGTATGGGCTCA
CCATATGTTTACAGTTGGGCTTTCTTACAACGCACTTATATATTTTACTGCCGGAACAATGATTATCGCAATTCCAACAGGTAT
CAAAATATTTAGCTGGATCGCAACTATGTGGGGTGGATCGATTACTTTCCCAACGCCTATGCTATTCTCTATAGGATTTATTAT
ATTATTCACGATTGGCGGCGTAACTGGCATAATCTTATCAAACTCGGCACTTGATAGAGTTCTGCACGATACATATTATGTTGT
GGCACATTTCCATTATACGATGTCGCTCGGTGCTTTATTCACTGCATTTGCCGGCTTTTATTATTGGTTCGGTAAAATATCAGG
CAAGCAATATCCCGAAATCTTAGGCAAAATCCATTTCTGGATTACTTTTGTCGGTGTTAATCTAACTTTCTTTAAAAAAGCTGG
AAGATGCTGGGGCAATGGATTATACAATCATTGTTTCGGCTACTGCATCGGAAGCTGCTGCATTACAATTTGTTGCTCCTTATG
CCGCATGTAGTATGGGTGAGTATTTCCGTGATAACGGCAAGCATGCACTTATTATTTATGATGATTTAAGTAAACATGCTGTCG
CATATAGACAAATTTCATTGTTACTTAGAAGACCGCCCGGACGTGAAGCATATCCCGGTGACGTATTTTACTTGCATTCAAGAT
TACTTGAGTGTGCTGCTAAAATGTCAGAGGAGAAAGGCGGCGGTTCACTTACGGCACTTCCTATAATCGAAACCCAAGCAGGTG
ACGTATCTGCTTATATTCCAACAAACGTTATTTCTATTACTGACGGTCAAATTTTCTTAGAAAGCGAGCTGTTTTATAAAGGTA
TAAGACCGGCTGTTAATGGATGGTTACTTCAAATGGTAGTGCTAATGTTTCTGGTATGGATATTGAATTAATTAATGCTCTTGC
TGCAAAAATAGGTATTAATATCGAGTACCACCAAGATAATTGGTATCAAGATCAGTTAGATATTCAAAGCGGTGTTGCTGACAT

>CA13-17-Dermacentor_variabilis_USA
TAACAAAAGCATTAGTGATTTTAATAAAAGATTTTCCGGTCTTTCTTTCGGTAGTCAAAAAAAGGAAGCTAGTTAATTTCTATA
ATCGGGTGGAGACATTTCCGCATAGATCAAGAACGTTTCCAGTGTGTCATCACGTTGGCGGCTTTTTTACGTGGATCGAGAGTC
GTCATTGCGAGTGAATGAAATGAGCGTGGCAATCCAGAAAATAATTAAAAATACTAATAAAATTAGTATTTTTAATTGGATCCC
CTGAATAAATCACGGGATGATAGGGGGAGAATGATCCACGTAACACACATTACTATGGGGTAAAAACTTACTCTAATCATTTAT
GTCGCTAATCTTTGTTTATAATATTAATAAACAAAGTCATCAAATTTCTTTATTGCGGGGTGGAGCAGCTCGGTAGCTATAACT
TATATAGATGGTGATCAAGGAATTTTGCGGCATCGTGGTTATGATATAAAAGATTTAGCCGAAAAAAGCGACTTTTTAGAGGTG
GCATATTTATTGATTTATGGGGAATTACCAAATAATAAGCAGTATAATGATTTTACTAAAAAGGTTGCTCATCATGCGTTAGTT
AATGAAAGATTACATTATTTATTCCAAACGTTTTGTAGCTCTTCGCATCCTATGGCTATTATGCTTGCGGCGGTTGGTTCTCTT
TCTGCATTTTATCCTGATTTGCTGAATTTTTTTAAAGAAGCGGATTATGAACTTACAGCTATTAGAATGATAGCTAAAATACCA
ACTATTGCTGCAATGGACACTCTACCCTCCTTTAAGTAGTATAAGCGGACATCCAGGAGCAGCGGTTGATATGGCTATTTTCAG
TCTGCATTTAACCGGTCTTTCATCAATACTTGGCTCAATCAACTTAATCGTTACTATCTTTAATATGAGAGCACCCGGGATGGG
ACTATTCAAAATGCCGTTATTTGTCTGGTCTATTTTAGTTACTGCATTCTTGATAATTTTAGCTATGCCAGTGCTTGGCGGAGC
TATTACTATGTTACTTACAGATCGTAATTTCGGTACTACTTTCTTTAAAACTGATGGTGGTGGTGATCCAGTATTATTTCAGCA
CTTATTTTGGTTTTTTGGTCACCCTGAAGTATATATTGTAATACTTCCAGGTTTTGGTATTGTAAGCCAAGTTATTTCCACTTT
CTCACGTAAACCAATATTTGGCTATCAAGGCATGGTTGGAGCCATGGTAATAATCGGCTTTGTCGGGTTTATTGTATGGGCTCA
CCATATGTTTACAGTTGGGCTTTCTTACAACGCACTTATATATTTTACTGCCGGAACAATGATTATCGCAATTCCAACAGGTAT
CAAAATATTTAGCTGGATCGCAACTATGTGGGGTGGATCGATTACTTTCCCAACGCCTATGCTATTCTCTATAGGATTTATTAT
ATTATTCACGATTGGCGGCGTAACTGGCATAATCTTATCAAACTCGGCACTTGATAGAGTTCTGCACGATACATATTATGTTGT
GGCACATTTCCATTATACGATGTCGCTCGGTGCTTTATTCACTGCATTTGCCGGCTTTTATTATTGGTTCGGTAAAATATCAGG
CAAGCAATATCCCGAAATCTTAGGCAAAATCCATTTCTGGATTACTTTTGTCGGTGTTAATCTAACTTTCTTTAAAAAAGCTGG
AAGATGCTGGGGCAATGGATTATACAATCATTGTTTCGGCTACTGCATCGGAAGCTGCTGCATTACAATTTGTTGCTCCTTATG
CCGCATGTAGTATGGGTGAGTATTTCCGTGATAACGGCAAGCATGCACTTATTATTTATGATGATTTAAGTAAACATGCTGTCG
CATATAGACAAATTTCATTGTTACTTAGAAGACCGCCCGGACGTGAAGCATATCCCGGTGACGTATTTTACTTGCATTCAAGAT
TACTTGAGTGTGCTGCTAAAATGTCAGAGGAGAAAGGCGGCGGTTCACTTACGGCACTTCCTATAATCGAAACCCAAGCAGGTG
ACGTATCTGCTTATATTCCAACAAACGTTATTTCTATTACTGACGGTCAAATTTTCTTAGAAAGCGAGCTGTTTTATAAAGGTA
TAAGACCGGCTGTTAATGGATGGTTACTTCAAATGGTAGTGCTAATGTTTCTGGTATGGATATTGAATTAATTAATGCTCTTGC
TGCAAAAATAGGTATTAATATCGAGTACCACCAAGATAATTGGTATCAAGATCAGTTAGATATTCAAAGCGGTGTTGCTGACAT
>Putah_Creek-_Dermacentor_variabilis_USA
TAACAAAAGCATTAGTGATTTTAATAAAAGATTTTCCGGTCTTTCTTTCGGTAGTCAAAAAAAGGAAGCTAGTTAATTTCTATA
ATCGGGTGGAGACATTTCCGCATAGATCAAGAACGTTTCCAGTGTGTCATCACGTTGGCGGCTTTTTTACGTGGATCGAGAGTC
GTCATTGCGAGTGAATGAAATGAGCGTGGCAATCCAGAAAATAATTAAAAATACTAATAAAATTAGTATTTTTAATTGGATCCC
CTGAATAAATCACGGGATGATAGGGGGAGAATGATCCACGTAACACACATTACTATGGGGTAAAAACTTACTCTAATCATTTAT
GTCGCTAATCTTTGTTTATAATATTAATAAACAAAGTCATCAAATTTCTTTATTGCGGGGTGGAGCAGCTCGGTAGCTATAACT
TATATAGATGGTGATCAAGGAATTTTGCGGCATCGTGGTTATGATATAAAAGATTTAGCCGAAAAAAGCGACTTTTTAGAGGTG
GCATATTTATTGATTTATGGGGAATTACCAAATAATAAGCAGTATAATGATTTTACTAAAAAGGTTGCTCATCATGCGTTAGTT
AATGAAAGATTACATTATTTATTCCAAACGTTTTGTAGCTCTTCGCATCCTATGGCTATTATGCTTGCGGCGGTTGGTTCTCTT
TCTGCATTTTATCCTGATTTGCTGAATTTTTTTAAAGAAGCGGATTATGAACTTACAGCTATTAGAATGATAGCTAAAATACCA
ACTATTGCTGCAATGGACACTCTACCCTCCTTTAAGTAGTATAAGCGGACATCCAGGAGCAGCGGTTGATATGGCTATTTTCAG
TCTGCATTTAACCGGTCTTTCATCAATACTTGGCTCAATCAACTTAATCGTTACTATCTTTAATATGAGAGCACCCGGGATGGG
ACTATTCAAAATGCCGTTATTTGTCTGGTCTATTTTAGTTACTGCATTCTTGATAATTTTAGCTATGCCAGTGCTTGGCGGAGC
TATTACTATGTTACTTACAGATCGTAATTTCGGTACTACTTTCTTTAAAACTGATGGTGGTGGTGATCCAGTATTATTTCAGCA
CTTATTTTGGTTTTTTGGTCACCCTGAAGTATATATTGTAATACTTCCAGGTTTTGGTATTGTAAGCCAAGTTATTTCCACTTT
CTCACGTAAACCAATATTTGGCTATCAAGGCATGGTTGGAGCCATGGTAATAATCGGCTTTGTCGGGTTTATTGTATGGGCTCA
CCATATGTTTACAGTTGGGCTTTCTTACAACGCACTTATATATTTTACTGCCGGAACAATGATTATCGCAATTCCAACAGGTAT
CAAAATATTTAGCTGGATCGCAACTATGTGGGGTGGATCGATTACTTTCCCAACGCCTATGCTATTCTCTATAGGATTTATTAT
ATTATTCACGATTGGCGGCGTAACTGGCATAATCTTATCAAACTCGGCACTTGATAGAGTTCTGCACGATACATATTATGTTGT
GGCACATTTCCATTATACGATGTCGCTCGGTGCTTTATTCACTGCATTTGCCGGCTTTTATTATTGGTTCGGTAAAATATCAGG
CAAGCAATATCCCGAAATCTTAGGCAAAATCCATTTCTGGATTACTTTTGTCGGTGTTAATCTAACTTTCTTTAAAAAAGCTGG
AAGATGCTGGGGCAATGGATTATACAATCATTGTTTCGGCTACTGCATCGGAAGCTGCTGCATTACAATTTGTTGCTCCTTATG
CCGCATGTAGTATGGGTGAGTATTTCCGTGATAACGGCAAGCATGCACTTATTATTTATGATGATTTAAGTAAACATGCTGTCG
CATATAGACAAATTTCATTGTTACTTAGAAGACCGCCCGGACGTGAAGCATATCCCGGTGACGTATTTTACTTGCATTCAAGAT
TACTTGAGTGTGCTGCTAAAATGTCAGAGGAGAAAGGCGGCGGTTCACTTACGGCACTTCCTATAATCGAAACCCAAGCAGGTG
ACGTATCTGCTTATATTCCAACAAACGTTATTTCTATTACTGACGGTCAAATTTTCTTAGAAAGCGAGCTGTTTTATAAAGGTA
TAAGACCGGCTGTTAATGGATGGTTACTTCAAATGGTAGTGCTAATGTTTCTGGTATGGATATTGAATTAATTAATGCTCTTGC
TGCAAAAATAGGTATTAATATCGAGTACCACCAAGATAATTGGTATCAAGATCAGTTAGATATTCAAAGCGGTGTTGCTGACAT
>Yolo-_Dermacentor_variabilis_USA
TAACAAAAGCATTAGTGATTTTAATAAAAGATTTTCCGGTCTTTCTTTCGGTAGTCAAAAAAAGGAAGCTAGTTAATTTCTATA
ATCGGGTGGAGACATTTCCGCATAGATCAAGAACGTTTCCAGTGTGTCATCACGTTGGCGGCTTTTTTACGTGGATCGAGAGTC
GTCATTGCGAGTGAATGAAATGAGCGTGGCAATCCAGAAAATAATTAAAAATACTAATAAAATTAGTATTTTTAATTGGATCCC
CTGAATAAATCACGGGATGATAGGGGGAGAATGATCCACGTAACACACATTACTATGGGGTAAAAACTTACTCTAATCATTTAT
GTCGCTAATCTTTGTTTATAATATTAATAAACAAAGTCATCAAATTTCTTTATTGCGGGGTGGAGCAGCTCGGTAGCTATAACT
TATATAGATGGTGATCAAGGAATTTTGCGGCATCGTGGTTATGATATAAAAGATTTAGCCGAAAAAAGCGACTTTTTAGAGGTG

GCATATTTATTGATTTATGGGGAATTACCAAATAATAAGCAGTATAATGATTTTACTAAAAAGGTTGCTCATCATGCGTTAGTT
AATGAAAGATTACATTATTTATTCCAAACGTTTTGTAGCTCTTCGCATCCTATGGCTATTATGCTTGCGGCGGTTGGTTCTCTT
TCTGCATTTTATCCTGATTTGCTGAATTTTTTTAAAGAAGCGGATTATGAACTTACAGCTATTAGAATGATAGCTAAAATACCA
ACTATTGCTGCAATGGACACTCTACCCTCCTTTAAGTAGTATAAGCGGACATCCAGGAGCAGCGGTTGATATGGCTATTTTCAG
TCTGCATTTAACCGGTCTTTCATCAATACTTGGCTCAATCAACTTAATCGTTACTATCTTTAATATGAGAGCACCCGGGATGGG
ACTATTCAAAATGCCGTTATTTGTCTGGTCTATTTTAGTTACTGCATTCTTGATAATTTTAGCTATGCCAGTGCTTGGCGGAGC
TATTACTATGTTACTTACAGATCGTAATTTCGGTACTACTTTCTTTAAAACTGATGGTGGTGGTGATCCAGTATTATTTCAGCA
CTTATTTTGGTTTTTTGGTCACCCTGAAGTATATATTGTAATACTTCCAGGTTTTGGTATTGTAAGCCAAGTTATTTCCACTTT
CTCACGTAAACCAATATTTGGCTATCAAGGCATGGTTGGAGCCATGGTAATAATCGGCTTTGTCGGGTTTATTGTATGGGCTCA
CCATATGTTTACAGTTGGGCTTTCTTACAACGCACTTATATATTTTACTGCCGGAACAATGATTATCGCAATTCCAACAGGTAT
CAAAATATTTAGCTGGATCGCAACTATGTGGGGTGGATCGATTACTTTCCCAACGCCTATGCTATTCTCTATAGGATTTATTAT
ATTATTCACGATTGGCGGCGTAACTGGCATAATCTTATCAAACTCGGCACTTGATAGAGTTCTGCACGATACATATTATGTTGT
GGCACATTTCCATTATACGATGTCGCTCGGTGCTTTATTCACTGCATTTGCCGGCTTTTATTATTGGTTCGGTAAAATATCAGG
CAAGCAATATCCCGAAATCTTAGGCAAAATCCATTTCTGGATTACTTTTGTCGGTGTTAATCTAACTTTCTTTAAAAAAGCTGG
AAGATGCTGGGGCAATGGATTATACAATCATTGTTTCGGCTACTGCATCGGAAGCTGCTGCATTACAATTTGTTGCTCCTTATG
CCGCATGTAGTATGGGTGAGTATTTCCGTGATAACGGCAAGCATGCACTTATTATTTATGATGATTTAAGTAAACATGCTGTCG
CATATAGACAAATTTCATTGTTACTTAGAAGACCGCCCGGACGTGAAGCATATCCCGGTGACGTATTTTACTTGCATTCAAGAT
TACTTGAGTGTGCTGCTAAAATGTCAGAGGAGAAAGGCGGCGGTTCACTTACGGCACTTCCTATAATCGAAACCCAAGCAGGTG
ACGTATCTGCTTATATTCCAACAAACGTTATTTCTATTACTGACGGTCAAATTTTCTTAGAAAGCGAGCTGTTTTATAAAGGTA
TAAGACCGGCTGTTAATGGATGGTTACTTCAAATGGTAGTGCTAATGTTTCTGGTATGGATATTGAATTAATTAATGCTCTTGC
TGCAAAAATAGGTATTAATATCGAGTACCACCAAGATAATTGGTATCAAGATCAGTTAGATATTCAAAGCGGTGTTGCTGACAT
>Stevenson_Bridge-_Dermacentor_variabilis_USA
TAACAAAAGCATTAGTGATTTTAATAAAAGATTTTCCGGTCTTTCTTTCGGTAGTCAAAAAAAGGAAGCTAGTTAATTTCTATA
ATCGGGTGGAGACATTTCCGCATAGATCAAGAACGTTTCCAGTGTGTCATCACGTTGGCGGCTTTTTTACGTGGATCGAGAGTC
GTCATTGCGAGTGAATGAAATGAGCGTGGCAATCCAGAAAATAATTAAAAATACTAATAAAATTAGTATTTTTAATTGGATCCC
CTGAATAAATCACGGGATGATAGGGGGAGAATGATCCACGTAACACACATTACTATGGGGTAAAAACTTACTCTAATCATTTAT
GTCGCTAATCTTTGTTTATAATATTAATAAACAAAGTCATCAAATTTCTTTATTGCGGGGTGGAGCAGCTCGGTAGCTATAACT
TATATAGATGGTGATCAAGGAATTTTGCGGCATCGTGGTTATGATATAAAAGATTTAGCCGAAAAAAGCGACTTTTTAGAGGTG
GCATATTTATTGATTTATGGGGAATTACCAAATAATAAGCAGTATAATGATTTTACTAAAAAGGTTGCTCATCATGCGTTAGTT
AATGAAAGATTACATTATTTATTCCAAACGTTTTGTAGCTCTTCGCATCCTATGGCTATTATGCTTGCGGCGGTTGGTTCTCTT
TCTGCATTTTATCCTGATTTGCTGAATTTTTTTAAAGAAGCGGATTATGAACTTACAGCTATTAGAATGATAGCTAAAATACCA
ACTATTGCTGCAATGGACACTCTACCCTCCTTTAAGTAGTATAAGCGGACATCCAGGAGCAGCGGTTGATATGGCTATTTTCAG
TCTGCATTTAACCGGTCTTTCATCAATACTTGGCTCAATCAACTTAATCGTTACTATCTTTAATATGAGAGCACCCGGGATGGG
ACTATTCAAAATGCCGTTATTTGTCTGGTCTATTTTAGTTACTGCATTCTTGATAATTTTAGCTATGCCAGTGCTTGGCGGAGC
TATTACTATGTTACTTACAGATCGTAATTTCGGTACTACTTTCTTTAAAACTGATGGTGGTGGTGATCCAGTATTATTTCAGCA
CTTATTTTGGTTTTTTGGTCACCCTGAAGTATATATTGTAATACTTCCAGGTTTTGGTATTGTAAGCCAAGTTATTTCCACTTT
CTCACGTAAACCAATATTTGGCTATCAAGGCATGGTTGGAGCCATGGTAATAATCGGCTTTGTCGGGTTTATTGTATGGGCTCA
CCATATGTTTACAGTTGGGCTTTCTTACAACGCACTTATATATTTTACTGCCGGAACAATGATTATCGCAATTCCAACAGGTAT
CAAAATATTTAGCTGGATCGCAACTATGTGGGGTGGATCGATTACTTTCCCAACGCCTATGCTATTCTCTATAGGATTTATTAT
ATTATTCACGATTGGCGGCGTAACTGGCATAATCTTATCAAACTCGGCACTTGATAGAGTTCTGCACGATACATATTATGTTGT
GGCACATTTCCATTATACGATGTCGCTCGGTGCTTTATTCACTGCATTTGCCGGCTTTTATTATTGGTTCGGTAAAATATCAGG
CAAGCAATATCCCGAAATCTTAGGCAAAATCCATTTCTGGATTACTTTTGTCGGTGTTAATCTAACTTTCTTTAAAAAAGCTGG
AAGATGCTGGGGCAATGGATTATACAATCATTGTTTCGGCTACTGCATCGGAAGCTGCTGCATTACAATTTGTTGCTCCTTATG
CCGCATGTAGTATGGGTGAGTATTTCCGTGATAACGGCAAGCATGCACTTATTATTTATGATGATTTAAGTAAACATGCTGTCG
CATATAGACAAATTTCATTGTTACTTAGAAGACCGCCCGGACGTGAAGCATATCCCGGTGACGTATTTTACTTGCATTCAAGAT
TACTTGAGTGTGCTGCTAAAATGTCAGAGGAGAAAGGCGGCGGTTCACTTACGGCACTTCCTATAATCGAAACCCAAGCAGGTG
ACGTATCTGCTTATATTCCAACAAACGTTATTTCTATTACTGACGGTCAAATTTTCTTAGAAAGCGAGCTGTTTTATAAAGGTA
TAAGACCGGCTGTTAATGGATGGTTACTTCAAATGGTAGTGCTAATGTTTCTGGTATGGATATTGAATTAATTAATGCTCTTGC
TGCAAAAATAGGTATTAATATCGAGTACCACCAAGATAATTGGTATCAAGATCAGTTAGATATTCAAAGCGGTGTTGCTGACAT
>OSU_83-1223-_Dermacentor_variabilis_USA
TAACAAAAGCATTAGTGATTTTAATAAAAGATTTTCCGGTCTTTCTTTCGGTAGTCAAAAAAAGGAAGCTAGTTAATTTCTATA
ATCGGGTGGAGACATTTCCGCATAGATCAAGAACGTTTCCAGTGTGTCATCACGTTGGCGGCTTTTTTACGTGGATCGAGAGTC
GTCATTGCGAGTGAATGAAATGAGCGTGGCAATCCAGAAAATAATTAAAAATACTAATTTTATTAGTATTTTTAATTGGATCCC
CTGAATAAATCACGGGATGATAGGGGGAGAATGATCCACGTAACACACATTACTATGGGGTAAAAACTTACTCTAATCATTTAT
GTCGCTAATCTTTGTTTATAATATTAATAAACAAAGTCATCAAATTTCTTTATTGCGGGGTGGAGCAGCTCGGTAGCTATAACT
TATATAGATGGTGATCAAGGAATTTTGCGGCATCGTGGTTATGATATAAAAGATTTAGCCGGAAAAAGCGACTTTTTAGAGGTG
GCATATTTATTGATTTATGGGGAATTACCAAATAATAAGCAGTATAATGATTTTACTAAAAAGGTTGCTCATCATGCGTTAGTT
AATGAAAGATTACATTATTTATTCCAAACGTTTTGTAGCTCTTCGCATCCTATGGCTATTATGCTTGCGGCGGTTGGTTCTCTT
TCTGCATTTTATCCTGATTTGCTGAATTTTTTTAAAGAAGCGGATTATGAACTTACAGCTATTAGAATGATAGCTAAAATACCA
ACTATTGCTGCAATGGACACTCTACCCTCCTTTAAGTAGTATAAGCGGACATCCAGGAGCAGCGGTTGATATGGCTATTTTCAG
TCTGCATTTAACCGGTCTTTCATCAATACTTGGCTCAATCAACTTAATCGTTACTATCTTTAATATGAGAGCACCCGGGATGGG
ACTATTCAAAATGCCGTTATTTGTCTGGTCTATTTTAGTTACTGCATTCTTGATAATTTTAGCTATGCCAGTGCTTGGCGGAGC
TATTACTATGTTACTTACCGATCGTAATTTCGGTACTACTTTCTTTAAAACTGATGGTGGTGGTGATCCAGTATTATTTCAGCA

CTTATTTTGGTTTTTTGGTCACCCTGAAGTATATATTGTAATACTTCCAGGTTTTGGTATTGTAAGCCAAGTTATTTCCACTTT
CTCACGTAAACCAATATTTGGCTATCAAGGCATGGTTGGAGCCATGGTAATAATCGGCTTTGTCGGGTTTATTGTATGGGCTCA
CCATATGTTTACAGTTGGGCTTTCTTACAACGCACTTATATATTTTACTGCCGGAACAATGATTATCGCAATTCCAACAGGTAT
CAAAATATTTAGCTGGATCGCAACTATGTGGGGTGGATCGATTACTTTCCCAACGCCTATGCTATTCTCTATAGGATTTATTAT
ATTATTCACGATTGGCGGCGTAACTGGCATAATCTTATCAAACTCGGCACTTGATAGAGTTCTGCACGATACATATTATGTTGT
GGCACATTTCCATTATACGATGTCGCTCGGTGCTTTATTCACTGCATTTGCCGGCTTTTATTATTGGTTCGGTAAAATATCAGG
CAAGCAATATCCCGAAATCTTAGGCAAAATCCATTTCTGGATTACTTTTGTCGGTGTTAATCTAACTTTCTTTAAAAAAGCTGG
AAGATGCTGGGGCAATGGATTATACAATCATTGTTTCGGCTACTGCATCGGAAGCTGCTGCATTACAATTTGTTGCTCCTTATG
CCGCATGTAGTATGGGTGAGTATTTCCGTGATAACGGCAAGCATGCACTTATTATTTATGATGATTTAAGTAAACATGCTGTCG
CATATAGACAAATTTCATTGTTACTTAGAAGACCGCCCGGACGTGAAGCATATCCCGGTGACGTATTTTACTTGCATTCAAGAT
TACTTGAGTGTGCTGCTAAAATGTCAGAGGAGAAAGGCGGCGGTTCACTTACGGCACTTCCTATAATCGAAACCCAAGCAGGTG
ACGTATCTGCTTATATTCCAACAAACGTTATTTCTATTACTGACGGTCAAATTTTCTTAGAAAGCGAGCTGTTTTATAAAGGTA
TAAGACCGGCTGTTAATGGATGGTTACTTCAAATGGTAGTGCTAATGTTTCTGGTATGGATATTGAATTAATTAATGCTCTTGC
TGCAAAAATAGGTATTAATATCGAGTACCACCAAGATAATTGGTATCAAGATCAGTTAGATATTCAAAGCGGTGCTGCTGACAT
>TX15-1-_Dermacentor_parumapertus_USA
TAACAAAAGCATTAGTGATTTTAATAAAAGATTTTCCGGTCTTTCTTTCGGTAGTCAAAAAAAGGAAGCTAGTTAATTTCTATA
ATCGGGTGGAGACATTTCCGTATAGATCAAGAACGTTTCCAGTGTGTCATCACGTTGGCGGCTTTTTTACGTGGATCGAGAGTC
GTCATTGCGAGTGAATGAAATGAGCGTGGCAATCCAGAAAATAATTAAAAATACTAATTTTATTAGTATTTTTAATTGGATCCC
CTGAATAAATCACGGGATGATAGGGGGAGAATGATCCACGTAACACACATTACTATGGGGTAAAAACTTACTCTAATCATTTAT
GTCGCTAATCTTTGTTTATAATATTAATAAACAAAGTCATCAAATTTCTTTATTGCGGGGTGGAGCAGCTCGGTAGCTATAACT
TATATAGATGGTGATCAAGGAATTTTGCGGCATCGTGGTTATGATATAAAAGATTTAGCCGAAAAAAGCGACTTTTTAGAGGTG
GCATATTTATTGATTTATGGGGAATTACCAAATAATAAGCAGTATAATGATTTTACTAAAAAGGTTGCTCATCATGCGTTAGTT
AATGAAAGATTACATTATTTATTCCAAACGTTTTGTAGCTCTTCGCATCCTATGGCTATTATGCTTGCGGCGGTTGGTTCTCTT
TCTGCATTTTATCCTGATTTGCTGAATTTTTTTAAAGAAGCGGATTATGAACTTACAGCTATTAGAATGATAGCTAAAATACCA
ACTATTGCTGCAATGGACACTCTACCCTCCTTTAAGTAGTATAAGCGGACATCCAGGAGCAGCGGTTGATATGGCTATTTTCAG
TCTGCATTTAACCGGTCTTTCATCAATACTTGGCTCAATCAACTTAATCGTTACTATCTTTAATATGAGAGCACCCGGGATGGG
ACTATTCAAAATGCCGTTATTTGTCTGGTCTATTTTAGTTACTGCATTCTTGATAATTTTAGCTATGCCAGTGCTTGGCGGAGC
TATTACTATGTTACTTACCGATCGTAATTTCGGTACTACTTTCTTTAAAACTGATGGTGGTGGTGATCCAGTATTATTTCAGCA
CTTATTTTGGTTTTTTGGTCACCCTGAAGTATATATTGTAATACTTCCAGGTTTTGGTATTGTAAGCCAAGTTATTTCCACTTT
CTCACGTAAACCAATATTTGGCTATCAAGGCATGGTTGGAGCCATGGTAATAATCGGCTTTGTCGGGTTTATTGTATGGGCTCA
CCATATGTTTACAGTTGGGCTTTCTTACAACGCACTTATATATTTTACTGCCGGAACAATGATTATCGCAATTCCAACAGGTAT
CAAAATATTTAGCTGGATCGCAACTATGTGGGGTGGATCGATTACTTTCCCAACGCCTATGCTATTCTCTATAGGATTTATTAT
ATTATTCACGATTGGCGGCGTAACTGGCATAATCTTATCAAACTCGGCACTTGATAGAGTTCTGCACGATACATATTATGTTGT
GGCACATTTCCATTATACGATGTCGCTCGGTGCTTTATTCACTGCATTTGCCGGCTTTTATTATTGGTTCGGTAAAATATCAGG
CAAGCAATATCCCGAAATCTTAGGCAAAATCCATTTCTGGATTACTTTTGTCGGTGTTAATCTAACTTTCTTTAAAAAAGCTGG
AAGATGCTGGGGCAATGGATTATACAATCATTGTTTCGGCTACTGCATCGGAAGCTGCTGCATTACAATTTGTTGCTCCTTATG
CCGCATGTAGTATGGGTGAGTATTTCCGTGATAACGGCAAGCATGCACTTATTATTTATGATGATTTAAGTAAACATGCTGTCG
CATATAGACAAATTTCATTGTTACTTAGAAGACCGCCCGGACGTGAAGCATATCCCGGTGACGTATTTTACTTGCATTCAAGAT
TACTTGAGTGTGCTGCTAAAATGTCAGAGGAGAAAGGCGGCGGTTCACTTACGGCACTTCCTATAATCGAAACCCAAGCAGGTG
ACGTATCTGCTTATATTCCAACAAACGTTATTTCTATTACTGACGGTCAAATTTTCTTAGAAAGCGAGCTGTTTTATAAAGGTA
TAAGACCGGCTGTTAATGGATGGTTACTTCAAATGGTAGTGCTAATGTTTCTGGTATGGATATTGAATTAATTAATGCTCTTGC
TGCAAAAATAGGTATTAATATCGAGTACCACCAAGATAATTGGTATCAAGATCAGTTAGATATTCAAAGCGGTGCTGCTGACAT
>OSU_83-117-_Dermacentor_variabilis_USA
TAACAAAAGCATTAGTGATTTTAATAAAAGATTTTCCGGTCTTTCTTTCGGTAGTCAAAAAAAGGAAGCTAGTTAATTTCTATA
ATCGGGTGGAGACATTTCCGCATAGATCAAGAACGTTTCCAGTGTGTCATCACGTTGGCGGCTTTTTTACGTGGATCGAGAGTC
GTCATTGCGAGTGAATGAAATGAGCGTGGCAATCCAGAAAATAATTAAAAATACTAATTTTATTAGTATTTTTAATTGGATCCC
CTGAATAAATCACGGGATGATAGGGGGAGAATGATCCACGTAACACACATTACTATGGGGTAAAAACTTACTCTAATCATTTAT
GTCGCTAATCTTTGTTTATAATATTAATAAACAAAGTCATCAAATTTCTTTATTGCGGGGTGGAGCAGCTCGGTAGCTATAACT
TATATAGATGGTGATCAAGGAATTTTGCGGCATCGTGGTTATGATATAAAAGATTTAGCCGGAAAAAGCGACTTTTTAGAGGTG
GCATATTTATTGATTTATGGGGAATTACCAAATAATAAGCAGTATAATGATTTTACTAAAAAGGTTGCTCATCATGCGTTAGTT
AATGAAAGATTACATTATTTATTCCAAACGTTTTGTAGCTCTTCGCATCCTATGGCTATTATGCTTGCGGCGGTTGGTTCTCTT
TCTGCATTTTATCCTGATTTGCTGAATTTTTTTAAAGAAGCGGATTATGAACTTACAGCTATTAGAATGATAGCTAAAATACCA
ACTATTGCTGCAATGGACACTCTACCCTCCTTTAAGTAGTATAAGCGGACATCCAGGAGCAGCGGTTGATATGGCTATTTTCAG
TCTGCATTTAACCGGTCTTTCATCAATACTTGGCTCAATCAACTTAATCGTTACTATCTTTAATATGAGAGCACCCGGGATGGG
ACTATTCAAAATGCCGTTATTTGTCTGGTCTATTTTAGTTACTGCATTCTTGATAATTTTAGCTATGCCAGTGCTTGGCGGAGC
TATTACTATGTTACTTACCGATCGTAATTTCGGTACTACTTTCTTTAAAACTGATGGTGGTGGTGATCCAGTATTATTTCAGCA
CTTATTTTGGTTTTTTGGTCACCCTGAAGTATATATTGTAATACTTCCAGGTTTTGGTATTGTAAGCCAAGTTATTTCCACTTT
CTCACGTAAACCAATATTTGGCTATCAAGGCATGGTTGGAGCCATGGTAATAATCGGCTTTGTCGGGTTTATTGTATGGGCTCA
CCATATGTTTACAGTTGGGCTTTCTTACAACGCACTTATATATTTTACTGCCGGAACAATGATTATCGCAATTCCAACAGGTAT
CAAAATATTTAGCTGGATCGCAACTATGTGGGGTGGATCGATTACTTTCCCAACGCCTATGCTATTCTCTATAGGATTTATTAT
ATTATTCACGATTGGCGGCGTAACTGGCATAATCTTATCAAACTCGGCACTTGATAGAGTTCTGCACGATACATATTATGTTGT
GGCACATTTCCATTATACGATGTCGCTCGGTGCTTTATTCACTGCATTTGCCGGCTTTTATTATTGGTTCGGTAAAATATCAGG
CAAGCAATATCCCGAAATCTTAGGCAAAATCCATTTCTGGATTACTTTTGTCGGTGTTAATCTAACTTTCTTTAAAAAAGCTGG

AAGATGCTGGGGCAATGGATTATACAATCATTGTTTCGGCTACTGCATCGGAAGCTGCTGCATTACAATTTGTTGCTCCTTATG
CCGCATGTAGTATGGGTGAGTATTTCCGTGATAACGGCAAGCATGCACTTATTATTTATGATGATTTAAGTAAACATGCTGTCG
CATATAGACAAATTTCATTGTTACTTAGAAGACCGCCCGGACGTGAAGCATATCCCGGTGACGTATTTTACTTGCATTCAAGAT
TACTTGAGTGTGCTGCTAAAATGTCAGAGGAGAAAGGCGGCGGTTCACTTACGGCACTTCCTATAATCGAAACCCAAGCAGGTG
ACGTATCTGCTTATATTCCAACAAACGTTATTTCTATTACTGACGGTCAAATTTTCTTAGAAAGCGAGCTGTTTTATAAAGGTA
TAAGACCGGCTGTTAATGGATGGTTACTTCAAATGGTAGTGCTAATGTTTCTGGTATGGATATTGAATTAATTAATGCTCTTGC
TGCAAAAATAGGTATTAATATCGAGTACCACCAAGATAATTGGTATCAAGATCAGTTAGATATTCAAAGCGGTGCTGCTGACAT
>OSU_85-1299-_Dermacentor_variabilis_USA
TAACAAAAGCATTAGTGATTTTAATAAAAGATTTTCCGGTCTTTCTTTCGGTAGTCAAAAAAAGGAAGCTAGTTAATTTCTATA
ATCGGGTGGAGACATTTCCGCATAGATCAAGAACGTTTCCAGTGTGTCATCACGTTGGCGGCTTTTTTACGTGGATCGAGAGTC
GTCATTGCGAGTGAATGAAATGAGCGTGGCAATCCAGAAAATAATTAAAAATACTAATTTTATTAGTATTTTTAATTGGATCCC
CTGAATAAATCACGGGATGATAGGGGGAGAATGATCCACGTAACACACATTACTATGGGGTAAAAACTTACTCTAATCATTTAT
GTCGCTAATCTTTGTTTATAATATTAATAAACAAAGTCATCAAATTTCTTTATTGCGGGGTGGAGCAGCTCGGTAGCTATAACT
TATATAGATGGTGATCAAGGAATTTTGCGGCATCGTGGTTATGATATAAAAGATTTAGCCGGAAAAAGCGACTTTTTAGAGGTG
GCATATTTATTGATTTATGGGGAATTACCAAATAATAAGCAGTATAATGATTTTACTAAAAAGGTTGCTCATCATGCGTTAGTT
AATGAAAGATTACATTATTTATTCCAAACGTTTTGTAGCTCTTCGCATCCTATGGCTATTATGCTTGCGGCGGTTGGTTCTCTT
TCTGCATTTTATCCTGATTTGCTGAATTTTTTTAAAGAAGCGGATTATGAACTTACAGCTATTAGAATGATAGCTAAAATACCA
ACTATTGCTGCAATGGACACTCTACCCTCCTTTAAGTAGTATAAGCGGACATCCAGGAGCAGCGGTTGATATGGCTATTTTCAG
TCTGCATTTAACCGGTCTTTCATCAATACTTGGCTCAATCAACTTAATCGTTACTATCTTTAATATGAGAGCACCCGGGATGGG
ACTATTCAAAATGCCGTTATTTGTCTGGTCTATTTTAGTTACTGCATTCTTGATAATTTTAGCTATGCCAGTGCTTGGCGGAGC
TATTACTATGTTACTTACCGATCGTAATTTCGGTACTACTTTCTTTAAAACTGATGGTGGTGGTGATCCAGTATTATTTCAGCA
CTTATTTTGGTTTTTTGGTCACCCTGAAGTATATATTGTAATACTTCCAGGTTTTGGTATTGTAAGCCAAGTTATTTCCACTTT
CTCACGTAAACCAATATTTGGCTATCAAGGCATGGTTGGAGCCATGGTAATAATCGGCTTTGTCGGGTTTATTGTATGGGCTCA
CCATATGTTTACAGTTGGGCTTTCTTACAACGCACTTATATATTTTACTGCCGGAACAATGATTATCGCAATTCCAACAGGTAT
CAAAATATTTAGCTGGATCGCAACTATGTGGGGTGGATCGATTACTTTCCCAACGCCTATGCTATTCTCTATAGGATTTATTAT
ATTATTCACGATTGGCGGCGTAACTGGCATAATCTTATCAAACTCGGCACTTGATAGAGTTCTGCACGATACATATTATGTTGT
GGCACATTTCCATTATACGATGTCGCTCGGTGCTTTATTCACTGCATTTGCCGGCTTTTATTATTGGTTCGGTAAAATATCAGG
CAAGCAATATCCCGAAATCTTAGGCAAAATCCATTTCTGGATTACTTTTGTCGGTGTTAATCTAACTTTCTTTAAAAAAGCTGG
AAGATGCTGGGGCAATGGATTATACAATCATTGTTTCGGCTACTGCATCGGAAGCTGCTGCATTACAATTTGTTGCTCCTTATG
CCGCATGTAGTATGGGTGAGTATTTCCGTGATAACGGCAAGCATGCACTTATTATTTATGATGATTTAAGTAAACATGCTGTCG
CATATAGACAAATTTCATTGTTACTTAGAAGACCGCCCGGACGTGAAGCATATCCCGGTGACGTATTTTACTTGCATTCAAGAT
TACTTGAGTGTGCTGCTAAAATGTCAGAGGAGAAAGGCGGCGGTTCACTTACGGCACTTCCTATAATCGAAACCCAAGCAGGTG
ACGTATCTGCTTATATTCCAACAAACGTTATTTCTATTACTGACGGTCAAATTTTCTTAGAAAGCGAGCTGTTTTATAAAGGTA
TAAGACCGGCTGTTAATGGATGGTTACTTCAAATGGTAGTGCTAATGTTTCTGGTATGGATATTGAATTAATTAATGCTCTTGC
TGCAAAAATAGGTATTAATATCGAGTACCACCAAGATAATTGGTATCAAGATCAGTTAGATATTCAAAGCGGTGCTGCTGACAT
>OSU_83-452-_Dermacentor_variabilis_USA
TAACAAAAGCATTAGTGATTTTAATAAAAGATTTTCCGGTCTTTCTTTCGGTAGTCAAAAAAAGGAAGCTAGTTAATTTCTATA
ATCGGGTGGAGACATTTCCGCATAGATCAAGAACGTTTCCAGTGTGTCATCACGTTGGCGGCTTTTTTACGTGGATCGAGAGTC
GTCATTGCGAGTGAATGAAATGAGCGTGGCAATCCAGAAAATAATTAAAAATACTAATTTTATTAGTATTTTTAATTGGATCCC
CTGAATAAATCACGGGATGATAGGGGGAGAATGATCCACGTAACACACATTACTATGGGGTAAAAACTTACTCTAATCATTTAT
GTCGCTAATCTTTGTTTATAATATTAATAAACAAAGTCATCAAATTTCTTTATTGCGGGGTGGAGCAGCTCGGTAGCTATAACT
TATATAGATGGTGATCAAGGAATTTTGCGGCATCGTGGTTATGATATAAAAGATTTAGCCGGAAAAAGCGACTTTTTAGAGGTG
GCATATTTATTGATTTATGGGGAATTACCAAATAATAAGCAGTATAATGATTTTACTAAAAAGGTTGCTCATCATGCGTTAGTT
AATGAAAGATTACATTATTTATTCCAAACGTTTTGTAGCTCTTCGCATCCTATGGCTATTATGCTTGCGGCGGTTGGTTCTCTT
TCTGCATTTTATCCTGATTTGCTGAATTTTTTTAAAGAAGCGGATTATGAACTTACAGCTATTAGAATGATAGCTAAAATACCA
ACTATTGCTGCAATGGACACTCTACCCTCCTTTAAGTAGTATAAGCGGACATCCAGGAGCAGCGGTTGATATGGCTATTTTCAG
TCTGCATTTAACCGGTCTTTCATCAATACTTGGCTCAATCAACTTAATCGTTACTATCTTTAATATGAGAGCACCCGGGATGGG
ACTATTCAAAATGCCGTTATTTGTCTGGTCTATTTTAGTTACTGCATTCTTGATAATTTTAGCTATGCCAGTGCTTGGCGGAGC
TATTACTATGTTACTTACCGATCGTAATTTCGGTACTACTTTCTTTAAAACTGATGGTGGTGGTGATCCAGTATTATTTCAGCA
CTTATTTTGGTTTTTTGGTCACCCTGAAGTATATATTGTAATACTTCCAGGTTTTGGTATTGTAAGCCAAGTTATTTCCACTTT
CTCACGTAAACCAATATTTGGCTATCAAGGCATGGTTGGAGCCATGGTAATAATCGGCTTTGTCGGGTTTATTGTATGGGCTCA
CCATATGTTTACAGTTGGGCTTTCTTACAACGCACTTATATATTTTACTGCCGGAACAATGATTATCGCAATTCCAACAGGTAT
CAAAATATTTAGCTGGATCGCAACTATGTGGGGTGGATCGATTACTTTCCCAACGCCTATGCTATTCTCTATAGGATTTATTAT
ATTATTCACGATTGGCGGCGTAACTGGCATAATCTTATCAAACTCGGCACTTGATAGAGTTCTGCACGATACATATTATGTTGT
GGCACATTTCCATTATACGATGTCGCTCGGTGCTTTATTCACTGCATTTGCCGGCTTTTATTATTGGTTCGGTAAAATATCAGG
CAAGCAATATCCCGAAATCTTAGGCAAAATCCATTTCTGGATTACTTTTGTCGGTGTTAATCTAACTTTCTTTAAAAAAGCTGG
AAGATGCTGGGGCAATGGATTATACAATCATTGTTTCGGCTACTGCATCGGAAGCTGCTGCATTACAATTTGTTGCTCCTTATG
CCGCATGTAGTATGGGTGAGTATTTCCGTGATAACGGCAAGCATGCACTTATTATTTATGATGATTTAAGTAAACATGCTGTCG
CATATAGACAAATTTCATTGTTACTTAGAAGACCGCCCGGACGTGAAGCATATCCCGGTGACGTATTTTACTTGCATTCAAGAT
TACTTGAGTGTGCTGCTAAAATGTCAGAGGAGAAAGGCGGCGGTTCACTTACGGCACTTCCTATAATCGAAACCCAAGCAGGTG
ACGTATCTGCTTATATTCCAACAAACGTTATTTCTATTACTGACGGTCAAATTTTCTTAGAAAGCGAGCTGTTTTATAAAGGTA
TAAGACCGGCTGTTAATGGATGGTTACTTCAAATGGTAGTGCTAATGTTTCTGGTATGGATATTGAATTAATTAATGCTCTTGC
TGCAAAAATAGGTATTAATATCGAGTACCACCAAGATAATTGGTATCAAGATCAGTTAGATATTCAAAGCGGTGCTGCTGACAT

>Skull_Valley-Dermacentor_parumapertus_USA
TAACAAAAGCATTAGTGATTTTAATAAAAGATTTTCCGGTCTTTCTTTCGGTAGTCAAAAAAAGGAAGCTAGTTAATTTCTATA
ATCGGGTGGAGACATTTCCGCATAGATCAAGAACGTTTCCAGTGTGTCATCACGTTGGCGGCTTTTTTACGTGGATCGAGAGTC
GTCATTGCGAGTGAATGAAATGAGCGTGGCAATCCAGAAAATAATTAAAAATACTAATTTTATTAGTATTTTTAATTGGATCCC
CTGAATAAATCACGGGATGATAGGGGGAGAATGATCCACGTAACACACATTACTATGGGGTAAAAACTTACTCTAATCATTTAT
GTCGCTAATCTTTGTTTATAATATTAATAAACAAAGTCATCAAATTTCTTTATTGCGGGGTGGAGCAGCTCGGTAGCTATAACT
TATATAGATGGTGATCAAGGAATTTTGCGGCATCGTGGTTATGATATAAAAGATTTAGCCGAAAAAAGCGACTTTTTAGAGGTG
GCATATTTATTGATTTATGGGGAATTACCAAATAATAAGCAGTATAATGATTTTACTAAAAAGGTTGCTCATCATGCGTTAGTT
AATGAAAGATTACATTATTTATTCCAAACGTTTTGTAGCTCTTCGCATCCTATGGCTATTATGCTTGCGGCGGTTGGTTCTCTT
TCTGCATTTTATCCTGATTTGCTGAATTTTTTTAAAGAAGCGGATTATGAACTTACAGCTATTAGAATGATAGCTAAAATACCA
ACTATTGCTGCAATGGACACTCTACCCTCCTTTAAGTAGTATAAGCGGACATCCAGGAGCAGCGGTTGATATGGCTATTTTCAG
TCTGCATTTAACCGGTCTTTCATCAATACTTGGCTCAATCAACTTAATCGTTACTATCTTTAATATGAGAGCACCCGGGATGGG
ACTATTCAAAATGCCGTTATTTGTCTGGTCTATTTTAGTTACTGCATTCTTGATAATTTTAGCTATGCCAGTGCTTGGCGGAGC
TATTACTATGTTACTTACCGATCGTAATTTCGGTACTACTTTCTTTAAAACTGATGGTGGTGGTGATCCAGTATTATTTCAGCA
CTTATTTTGGTTTTTTGGTCACCCTGAAGTATATATTGTAATACTTCCAGGTTTTGGTATTGTAAGCCAAGTTATTTCCACTTT
CTCACGTAAACCAATATTTGGCTATCAAGGCATGGTTGGAGCCATGGTAATAATCGGCTTTGTCGGGTTTATTGTATGGGCTCA
CCATATGTTTACAGTTGGGCTTTCTTACAACGCACTTATATATTTTACTGCCGGAACAATGATTATCGCAATTCCAACAGGTAT
CAAAATATTTAGCTGGATCGCAACTATGTGGGGTGGATCGATTACTTTCCCAACGCCTATGCTATTCTCTATAGGATTTATTAT
ATTATTCACGATTGGCGGCGTAACTGGCATAATCTTATCAAACTCGGCACTTGATAGAGTTCTGCACGATACATATTATGTTGT
GGCACATTTCCATTATACGATGTCGCTCGGTGCTTTATTCACTGCATTTGCCGGCTTTTATTATTGGTTCGGTAAAATATCAGG
CAAGCAATATCCCGAAATCTTAGGCAAAATCCATTTCTGGATTACTTTTGTCGGTGTTAATCTAACTTTCTTTAAAAAAGCTGG
AAGATGCTGGGGCAATGGATTATACAATCATTGTTTCGGCTACTGCATCGGAAGCTGCTGCATTACAATTTGTTGCTCCTTATG
CCGCATGTAGTATGGGTGAGTATTTCCGTGATAACGGCAAGCATGCACTTATTATTTATGATGATTTAAGTAAACATGCTGTCG
CATATAGACAAATTTCATTGTTACTTAGAAGACCGCCCGGACGTGAAGCATATCCCGGTGACGTATTTTACTTGCATTCAAGAT
TACTTGAGTGTGCTGCTAAAATGTCAGAGGAGAAAGGCGGCGGTTCACTTACGGCACTTCCTATAATCGAAACCCAAGCAGGTG
ACGTATCTGCTTATATTCCAACAAACGTTATTTCTATTACTGACGGTCAAATTTTCTTAGAAAGCGAGCTGTTTTATAAAGGTA
TAAGACCGGCTGTTAATGGATGGTTACTTCAAATGGTAGTGCTAATGTTTCTGGTATGGATATTGAATTAATTAATGCTCTTGC
TGCAAAAATAGGTATTAATATCGAGTACCACCAAGATAATTGGTATCAAGATCAGTTAGATATTCAAAGCGGTGCTGCTGACAT
>OSU_85-389-_Dermacentor_variabilis_USA
TAACAAAAGCATTAGTGATTTTAATAAAAGATTTTCCGGTCTTTCTTTCGGTAGTCAAAAAAAGGAAGCTAGTTAATTTCTATA
ATCGGGTGGAGACATTTCCGCATAGATCAAGAACGTTTCCAGTGTGTCATCACGTTGGCGGCTTTTTTACGTGGATCGAGAGTC
GTCATTGCGAGTGAATGAAATGAGCGTGGCAATCCAGAAAATAATTAAAAATACTAATTTTATTAGTATTTTTAATTGGATCCC
CTGAATAAATCACGGGATGATAGGGGGAGAATGATCCACGTAACACACATTACTATGGGGTAAAAACTTACTCTAATCATTTAT
GTCGCTAATCTTTGTTTATAATATTAATAAACAAAGTCATCAAATTTCTTTATTGCGGGGTGGAGCAGCTCGGTAGCTATAACT
TATATAGATGGTGATCAAGGAATTTTGCGGCATCGTGGTTATGATATAAAAGATTTAGCCGGAAAAAGCGACTTTTTAGAGGTG
GCATATTTATTGATTTATGGGGAATTACCAAATAATAAGCAGTATAATGATTTTACTAAAAAGGTTGCTCATCATGCGTTAGTT
AATGAAAGATTACATTATTTATTCCAAACGTTTTGTAGCTCTTCGCATCCTATGGCTATTATGCTTGCGGCGGTTGGTTCTCTT
TCTGCATTTTATCCTGATTTGCTGAATTTTTTTAAAGAAGCGGATTATGAACTTACAGCTATTAGAATGATAGCTAAAATACCA
ACTATTGCTGCAATGGACACTCTACCCTCCTTTAAGTAGTATAAGCGGACATCCAGGAGCAGCGGTTGATATGGCTATTTTCAG
TCTGCATTTAACCGGTCTTTCATCAATACTTGGCTCAATCAACTTAATCGTTACTATCTTTAATATGAGAGCACCCGGGATGGG
ACTATTCAAAATGCCGTTATTTGTCTGGTCTATTTTAGTTACTGCATTCTTGATAATTTTAGCTATGCCAGTGCTTGGCGGAGC
TATTACTATGTTACTTACCGATCGTAATTTCGGTACTACTTTCTTTAAAACTGATGGTGGTGGTGATCCAGTATTATTTCAGCA
CTTATTTTGGTTTTTTGGTCACCCTGAAGTATATATTGTAATACTTCCAGGTTTTGGTATTGTAAGCCAAGTTATTTCCACTTT
CTCACGTAAACCAATATTTGGCTATCAAGGCATGGTTGGAGCCATGGTAATAATCGGCTTTGTCGGGTTTATTGTATGGGCTCA
CCATATGTTTACAGTTGGGCTTTCTTACAACGCACTTATATATTTTACTGCCGGAACAATGATTATCGCAATTCCAACAGGTAT
CAAAATATTTAGCTGGATCGCAACTATGTGGGGTGGATCGATTACTTTCCCAACGCCTATGCTATTCTCTATAGGATTTATTAT
ATTATTCACGATTGGCGGCGTAACTGGCATAATCTTATCAAACTCGGCACTTGATAGAGTTCTGCACGATACATATTATGTTGT
GGCACATTTCCATTATACGATGTCGCTCGGTGCTTTATTCACTGCATTTGCCGGCTTTTATTATTGGTTCGGTAAAATATCAGG
CAAGCAATATCCCGAAATCTTAGGCAAAATCCATTTCTGGATTACTTTTGTCGGTGTTAATCTAACTTTCTTTAAAAAAGCTGG
AAGATGCTGGGGCAATGGATTATACAATCATTGTTTCGGCTACTGCATCGGAAGCTGCTGCATTACAATTTGTTGCTCCTTATG
CCGCATGTAGTATGGGTGAGTATTTCCGTGATAACGGCAAGCATGCACTTATTATTTATGATGATTTAAGTAAACATGCTGTCG
CATATAGACAAATTTCATTGTTACTTAGAAGACCGCCCGGACGTGAAGCATATCCCGGTGACGTATTTTACTTGCATTCAAGAT
TACTTGAGTGTGCTGCTAAAATGTCAGAGGAGAAAGGCGGCGGTTCACTTACGGCACTTCCTATAATCGAAACCCAAGCAGGTG
ACGTATCTGCTTATATTCCAACAAACGTTATTTCTATTACTGACGGTCAAATTTTCTTAGAAAGCGAGCTGTTTTATAAAGGTA
TAAGACCGGCTGTTAATGGATGGTTACTTCAAATGGTAGTGCTAATGTTTCTGGTATGGATATTGAATTAATTAATGCTCTTGC
TGCAAAAATAGGTATTAATATCGAGTACCACCAAGATAATTGGTATCAAGATCAGTTAGATATTCAAAGCGGTGCTGCTGACAT
>CA-459-_Haemaphysalis_leporispalustrus_USA
TAACAAAAGCATTAGTGATTTTAATAAAAGATTTTCCGGTCTTTCTTTCGGTAGTCAAAAAAAGGAAGCTAGTTAATTTCTATA
ATCGGGTGGAGACATTTCCGCATAGATCAAGAACGTTTCCAGTGTGTCATCACGTTGGCGGCTTTTTTACGTGGATCGAGAGTC
GTCATTGCGAGTGAATGAAATGAGCGTGGCAATCCAGAAAATAATAAAAAATACTAATTTTATTAGTATTTTTAATTGGATCCC
CTGAATAAATCACGGGATGATAGGGGGAGAATGATCCACGTAACACACATTACTATGGGGTAAAAACTTACTCTAATCATTTAT
GTCGCTAATCTTTGTTTATAATATTAATAAACAAAGTCATCAAATTTCTTTATTGCGGGGTGGAGCAGCTCGGTAGCTATAACT
TATATAGATGGTGATCAAGGAATTTTGCGGCATCGTGGTTATGATATAAAAGATTTAGCCAAAAAAAGCGACTTTTTAGAGGTG

GCATATTTATTGATTTATGGGGAATTACCAAATAATAAGCAGTATAATGATTTTACTAAAAAGGTTGCTCATCATGCGTTAGTT
AATGAAAGATTACATTATTTATTCCAAACGTTTTGTAGCTCTTCGCATCCTATGGCTATTATGCTTGCGGCGGTTGGTTCTCTT
TCTGCATTTTATCCTGATTTGCTGAATTTTTTTAAAGAAGCGGATTATGAACTTACAGCTATTAGAATGATAGCTAAAATACCA
ACTATTGCTGCAATGGACACTCTACCCTCCTTTAAGTAGTATAAGCGGACATCCAGGAGCAGCGGTTGATATGGCTATTTTCAG
TCTGCATTTAACCGGTCTTTCATCAATACTTGGCTCAATCAACTTAATCGTTACTATCTTTAATATGAGAGCACCCGGGATGGG
ACTATTCAAAATGCCGTTATTTGTCTGGTCTATTTTAGTTACTGCATTCTTGATAATTTTAGCTATGCCAGTGCTTGGCGGAGC
TATTACTATGTTACTTACCGATCGTAATTTCGGTACTACTTTCTTTAAAACTGATGGTGGTGGTGATCCAGTATTATTTCAGCA
CTTATTTTGGTTTTTTGGTCACCCTGAAGTATATATTGTAATACTTCCAGGTTTTGGTATTGTAAGCCAAGTTATTTCCACTTT
CTCACGTAAACCAATATTTGGCTATCAAGGCATGGTTGGAGCCATGGTAATAATCGGCTTTGTCGGGTTTATTGTATGGGCTCA
CCATATGTTTACAGTTGGGCTTTCTTACAACGCACTTATATATTTTACTGCTGGAACAATGATTATCGCAATTCCAACAGGTAT
CAAAATATTTAGCTGGATCGCAACTATGTGGGGTGGATCGATTACTTTCCCAACGCCTATGCTATTCTCTATAGGATTTATTAT
ATTATTCACGATTGGCGGCGTAACTGGCATAATCTTATCAAACTCGGCACTTGATAGAGTTCTGCACGATACATATTATGTTGT
GGCACATTTCCATTATACGATGTCGCTCGGTGCTTTATTCACTGCATTTGCCGGCTTTTATTATTGGTTCGGTAAAATATCAGG
CAAGCAATATCCCGAAATCTTAGGCAAAATCCATTTCTGGATTACTTTTGTCGGTGTTAATCTAACTTTCTTTAAAAAAGCTGG
AAGATGCTGGGGCAATGGATTATACAATCATTGTTTCGGCTACTGCATCAGAAGCTGCTGCATTACAATTTGTTGCTCCTTATG
CCGCATGTAGTATGGGTGAGTATTTCCGTGATAACGGCAAGCATGCACTTATTATTTATGATGATTTAAGTAAACATGCTGTCG
CATATAGACAAATTTCATTGTTACTTAGAAGACCGCCCGGACGTGAAGCATATCCCGGTGACGTATTTTACTTGCATTCAAGAT
TACTTGAGTGTGCTGCTAAAATGTCAGAGGAGAAAGGCGGCGGTTCACTTACGGCACTTCCTATAATCGAAACCCAAGCAGGTG
ACGTATCTGCTTATATTCCAACAAACGTTATTTCTATTACTGACGGTCAAATTTTCTTAGAAAGCGAGCTGTTTTATAAAGGTA
TAAGACCGGCTGTTAATGGATGGTTACTTCAAATGGGAGTGCTAATGTTTCTGGTATGGATATTGAATTAATTAATGCTCTTGC
TGCAAAAATAGGTATTAATATCGAGTACCACCAAGATAATTGGTATCAAGATCAGTTAGATATTCAAAGCGGTGCTGCTGACAT

Supplementary Figure 1. Molecular phylogenetic analysis of 30 isolates of *Rickettsia bellii* from North and South America. A total of 2,269 aligned nucleotide sites of 3 protein coding genes (*glt*A, *atp*A, *cox*A) and 2 intergenic regions (*rpm*E-tRNA*fmet* , RC1027-*xth*A2) were concatenated and subjected to Bayesian analysis. A total of 10,000,000 generations were run using the GTR model with a sample frequency of 10,000. The analysis was run with 3 heated chains, and the first 1,000,000 generations were discarded as burnin. Numbers at nodes are support values derived from posterior probability. The scale bar is in units of expected substitutions per site.

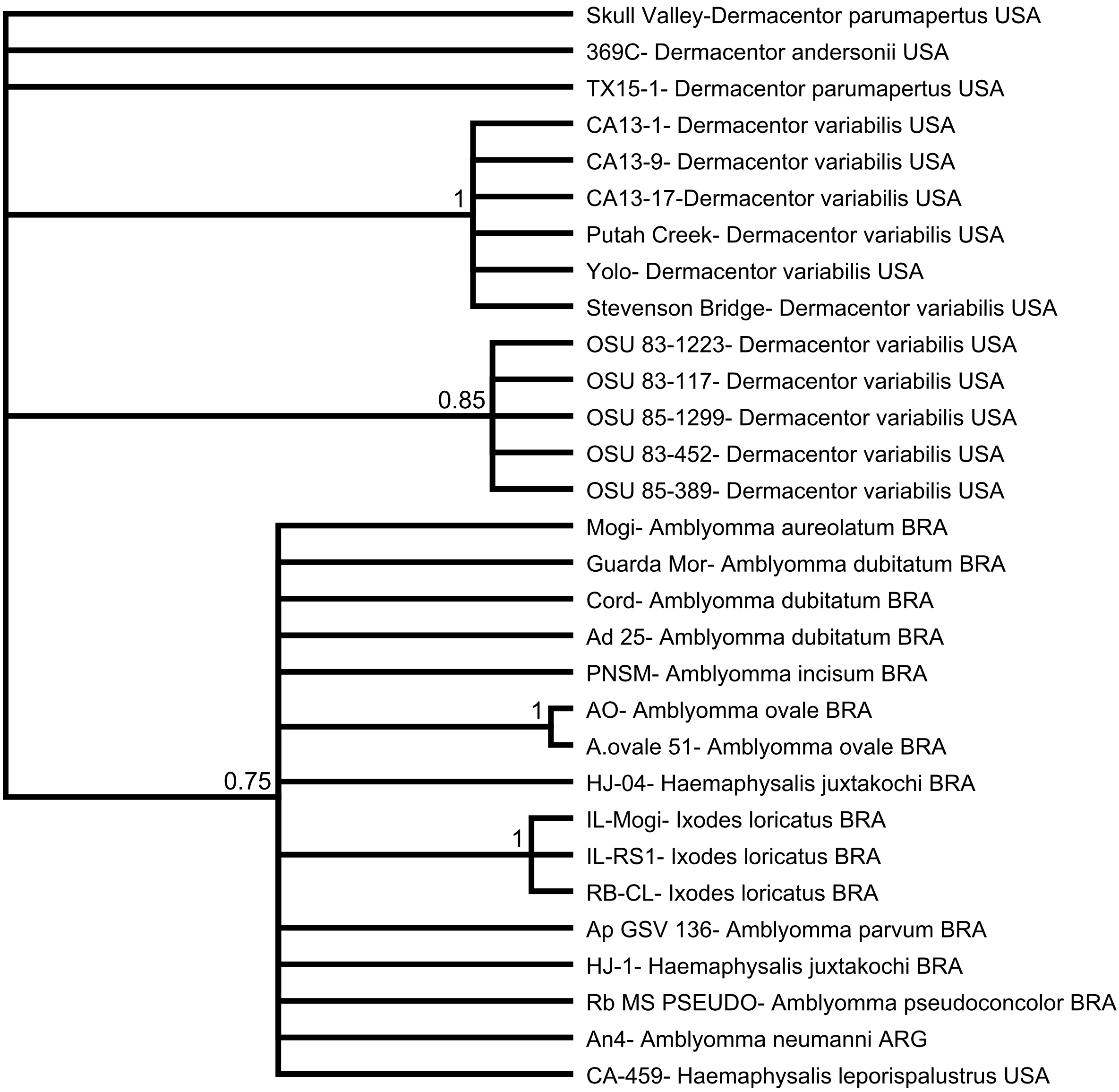
Skull Valley-Dermacentor parumapertus USA
369C- Dermacentor andersonii USA
TX15-1- Dermacentor parumapertus USA
CA13-1- Dermacentor variabilis USA
CA13-9- Dermacentor variabilis USA
CA13-17-Dermacentor variabilis USA
Putah Creek- Dermacentor variabilis USA
Yolo- Dermacentor variabilis USA
Stevenson Bridge- Dermacentor variabilis USA
OSU 83-1223- Dermacentor variabilis USA
OSU 83-117- Dermacentor variabilis USA
OSU 85-1299- Dermacentor variabilis USA
OSU 83-452- Dermacentor variabilis USA
OSU 85-389- Dermacentor variabilis USA
Mogi- Amblyomma aureolatum BRA
Guarda Mor- Amblyomma dubitatum BRA
Cord- Amblyomma dubitatum BRA
Ad 25- Amblyomma dubitatum BRA
PNSM- Amblyomma incisum BRA
AO- Amblyomma ovale BRA
A.ovale 51- Amblyomma ovale BRA
HJ-04- Haemaphysalis juxtakochi BRA
IL-Mogi- Ixodes loricatus BRA
IL-RS1- Ixodes loricatus BRA
RB-CL- Ixodes loricatus BRA
Ap GSV 136- Amblyomma parvum BRA
HJ-1- Haemaphysalis juxtakochi BRA
Rb MS PSEUDO- Amblyomma pseudoconcolor BRA
An4- Amblyomma neumanni ARG
CA-459- Haemaphysalis leporispalustrus USA
1
0.85
1
0.75
1

Supplementary Figure 2. Molecular phylogenetic analysis of 30 isolates of *Rickettsia bellii* from North and South America. A total of 2,269 aligned nucleotide sites of 3 protein coding genes (*glt*A, *atp*A, *cox*A) and 2 intergenic regions (*rpm*E-tRNA*fmet* , RC1027-*xth*A2) were concatenated and subjected to Bayesian analysis. A total of 10,000,000 generations were run using the HKY85 model with a sample frequency of 10,000. The analysis was run with 3 heated chains, and the first 1,000,000 generations were discarded as burnin. Numbers at nodes are support values derived from posterior probability. The scale bar is in units of expected substitutions per site.

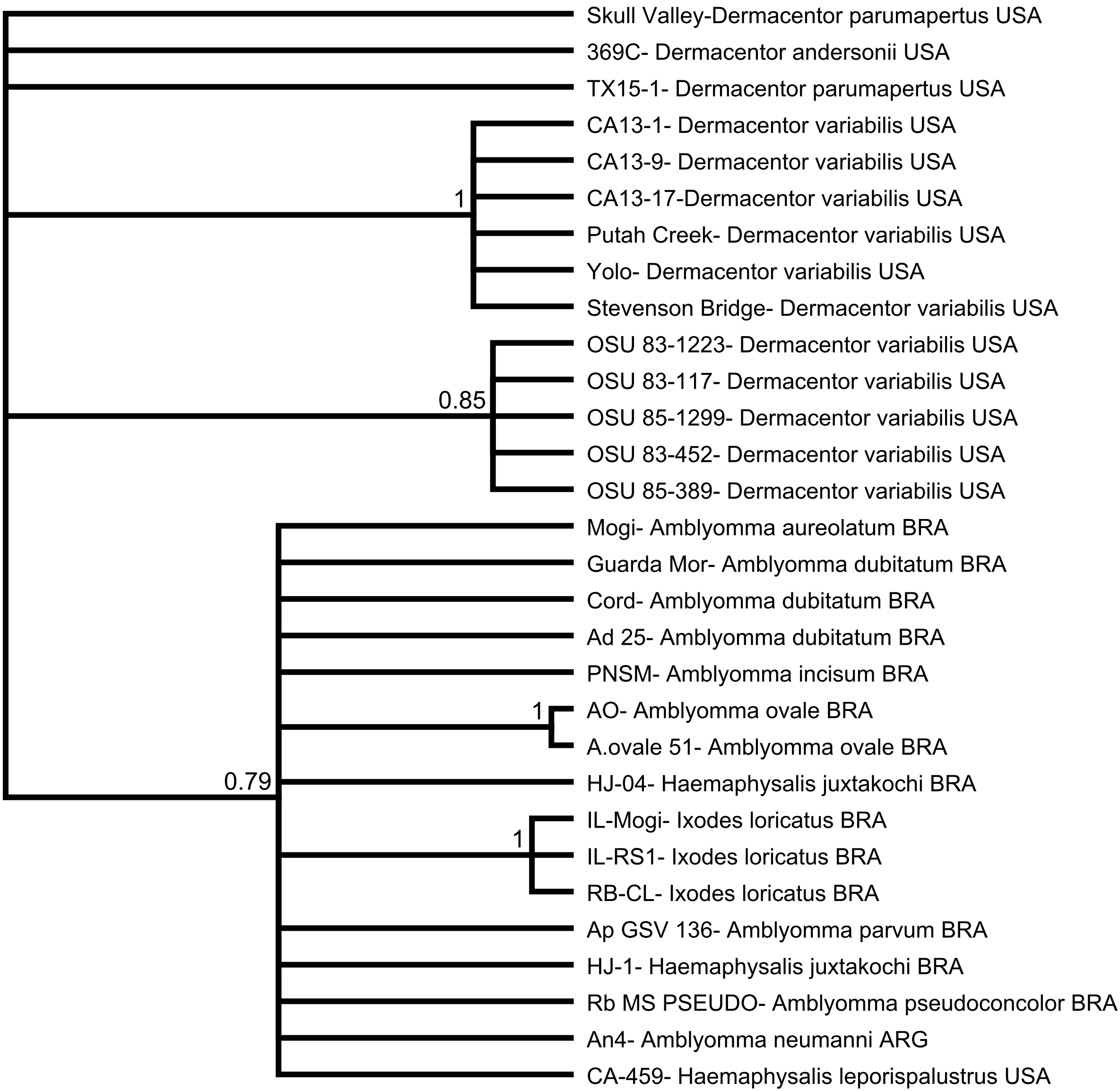
Skull Valley-Dermacentor parumapertus USA
369C- Dermacentor andersonii USA
TX15-1- Dermacentor parumapertus USA
CA13-1- Dermacentor variabilis USA
CA13-9- Dermacentor variabilis USA
1
CA13-17-Dermacentor variabilis USA
Putah Creek- Dermacentor variabilis USA
Yolo- Dermacentor variabilis USA
Stevenson Bridge- Dermacentor variabilis USA
OSU 83-1223- Dermacentor variabilis USA
OSU 83-117- Dermacentor variabilis USA
0.85
OSU 85-1299- Dermacentor variabilis USA
OSU 83-452- Dermacentor variabilis USA
OSU 85-389- Dermacentor variabilis USA
Mogi- Amblyomma aureolatum BRA
Guarda Mor- Amblyomma dubitatum BRA
Cord- Amblyomma dubitatum BRA
Ad 25- Amblyomma dubitatum BRA
PNSM- Amblyomma incisum BRA
1
AO- Amblyomma ovale BRA
A.ovale 51- Amblyomma ovale BRA
0.79
HJ-04- Haemaphysalis juxtakochi BRA
IL-Mogi- Ixodes loricatus BRA
1
IL-RS1- Ixodes loricatus BRA
RB-CL- Ixodes loricatus BRA
Ap GSV 136- Amblyomma parvum BRA
HJ-1- Haemaphysalis juxtakochi BRA
Rb MS PSEUDO- Amblyomma pseudoconcolor BRA
An4- Amblyomma neumanni ARG
CA-459- Haemaphysalis leporispalustrus USA
