# Supplementary material for: Genotypic Characterization of Rickettsia bellii Reveals Distinct Lineages in the United States and South America
Source: Biomed Res Int. 2018 Apr 8;2018:8505483. doi: 10.1155/2018/8505483 (PMC5911327; doi:10.1155/2018/8505483)
Supplement: Supplementary Materials — An identity matrix table of the 2,269-nucleotide alignment used for the phylogeny if R. bellii is available in the supplemental material for this article (Table S1). The final alignment comprising 2,269 nucleotides was concatenated in the following order: rpmE-tRNAfmet (414-nt), gltA (357-nt), coxA (898-nt), atpA (449-nt), and RC1027-xthA2 (151-nt); and it is also available in the supplementary material (Rbellii final alignment.fas). Two additional phylogenetic trees are also available in the supplementary material (Rbellii GTR tree.pdf and Rbellii HKY tree.pdf). Table S1: identity matrix of a 2,269-nucleotide alignment of the 30 Rickettsia bellii isolates from Brazil (BRA), Argentina (ARG), and the United States (USA) used in the present study. Supplementary Figure 1: molecular phylogenetic analysis of 30 isolates of Rickettsia bellii from North and South America. A total of 2,269 aligned nucleotide sites of 3 protein coding genes (gltA, atpA, and coxA) and 2 intergenic regions (rpmE-tRNAfmet and RC1027-xthA2) were concatenated and subjected to Bayesian analysis. A total of 10,000,000 generations were run using the GTR model with a sample frequency of 10,000. The analysis was run with 3 heated chains, and the first 1,000,000 generations were discarded as burn-in. Numbers at nodes are support values derived from posterior probability. The scale bar is in units of expected substitutions per site. Supplementary Figure 2: molecular phylogenetic analysis of 30 isolates of Rickettsia bellii from North and South America. A total of 2,269 aligned nucleotide sites of 3 protein coding genes (gltA, atpA, and coxA) and 2 intergenic regions (rpmE-tRNAfmet and RC1027-xthA2) were concatenated and subjected to Bayesian analysis. A total of 10,000,000 generations were run using the HKY85 model with a sample frequency of 10,000. The analysis was run with 3 heated chains, and the first 1,000,000 generations were discarded as burn-in. Numbers at nodes are support values derived from p [file Table S1.pdf › Table S1.pdf]

Table S1. Identity matrix of a 2,269-nucleotide alignment of the 30 *Rickettsia bellii* isolates from Brazil (BRA), Argentina (ARG) and the United States (USA) used in the present study

	ISOLATES	1	2	3	4	5	6	7	8	9	10	11	12	13	14	15	16	17	18	19	20	21	22	23	24	25	26	27	28	29	30
1	Mogi- Amblyomma aureolatum BRA																														
2	Guarda Mor- Amblyomma dubitatum BRA	99.96	*																												
3	Ad-CORD-SP- Amblyomma dubitatum BRA	99.96	100	*																											
4	Ad 25- Amblyomma dubitatum BRA	99.96	100	100	*																										
5	PNSM- Amblyomma incisum BRA	99.96	100	100	100	*																									
6	AO- Amblyomma ovale BRA	99.82	99.87	99.87	99.87	99.87	*																								
7	HJ#4- Haemaphysalis juxtakochi BRA	99.96	100	100	100	100	99.87	*																							
8	IL-Mogi- Ixodes loricatus BRA	99.82	99.87	99.87	99.87	99.87	99.74	99.87	*																						
9	Ap GSV 136- Amblyomma parvum BRA	99.91	99.96	99.96	99.96	99.96	99.82	99.96	99.82	*																					
10	IL-RS1- Ixodes loricatus BRA	99.82	99.87	99.87	99.87	99.87	99.74	99.87	100	99.82	*																				
11	RB-CL- Ixodes loricatus BRA	99.82	99.87	99.87	99.87	99.87	99.74	99.87	100	99.82	100	*																			
12	HJ#1- Haemaphysalis juxtakochi BRA	99.96	100	100	100	100	99.87	100	99.87	99.96	99.87	99.87	*																		
13	Rb MS PSEUDO- Amblyomma pseudoconcolor BRA	99.87	99.91	99.91	99.91	99.91	99.78	99.91	99.78	99.87	99.78	99.78	99.91	*																	
14	P-10 Amblyomma ovale BRA	99.82	99.87	99.87	99.87	99.87	100	99.87	99.74	99.82	99.74	99.74	99.87	99.78	*																
15	An4- Amblyomma neumanni ARG	99.91	99.96	99.96	99.96	99.96	99.82	99.96	99.82	99.91	99.82	99.82	99.96	99.87	99.82	*															
16	369C- Dermacentor andersonii USA	99.82	99.87	99.87	99.87	99.87	99.74	99.87	99.74	99.91	99.74	99.74	99.87	99.78	99.74	99.82	*														
17	CA13-1- Dermacentor variabilis USA	99.69	99.74	99.74	99.74	99.74	99.6	99.74	99.6	99.69	99.6	99.6	99.74	99.65	99.6	99.69	99.69	*													
18	CA13-9- Dermacentor variabilis USA	99.69	99.74	99.74	99.74	99.74	99.6	99.74	99.6	99.69	99.6	99.6	99.74	99.65	99.6	99.69	99.69	100	*												
19	CA13-17- Dermacentor variabilis USA	99.69	99.74	99.74	99.74	99.74	99.6	99.74	99.6	99.69	99.6	99.6	99.74	99.65	99.6	99.69	99.69	100	100	*											
20	Putah Creek- Dermacentor variabilis USA	99.69	99.74	99.74	99.74	99.74	99.6	99.74	99.6	99.69	99.6	99.6	99.74	99.65	99.6	99.69	99.69	100	100	100	*										
21	Yolo- Dermacentor variabilis USA	99.69	99.74	99.74	99.74	99.74	99.6	99.74	99.6	99.69	99.6	99.6	99.74	99.65	99.6	99.69	99.69	100	100	100	100	*									
22	Stevenson Bridge- Dermacentor variabilis USA	99.69	99.74	99.74	99.74	99.74	99.6	99.74	99.6	99.69	99.6	99.6	99.74	99.65	99.6	99.69	99.69	100	100	100	100	100	*								
23	OSU 83-1223- Dermacentor variabilis USA	99.87	99.91	99.91	99.91	99.91	99.78	99.91	99.87	99.87	99.87	99.87	99.91	99.82	99.78	99.87	99.87	99.74	99.74	99.74	99.74	99.74	99.74	*							
24	TX15-1- Dermacentor parumapertus USA	99.87	99.91	99.91	99.91	99.91	99.78	99.91	99.78	99.87	99.78	99.78	99.91	99.82	99.78	99.87	99.87	99.74	99.74	99.74	99.74	99.74	99.74	99.91	*						
25	OSU 83-117- Dermacentor variabilis USA	99.87	99.91	99.91	99.91	99.91	99.78	99.91	99.87	99.87	99.87	99.87	99.91	99.82	99.78	99.87	99.87	99.74	99.74	99.74	99.74	99.74	99.74	100	99.91	*					
26	OSU 85-1299- Dermacentor variabilis USA	99.87	99.91	99.91	99.91	99.91	99.78	99.91	99.87	99.87	99.87	99.87	99.91	99.82	99.78	99.87	99.87	99.74	99.74	99.74	99.74	99.74	99.74	100	99.91	100	*				
27	OSU 83-452- Dermacentor variabilis USA	99.87	99.91	99.91	99.91	99.91	99.78	99.91	99.87	99.87	99.87	99.87	99.91	99.82	99.78	99.87	99.87	99.74	99.74	99.74	99.74	99.74	99.74	100	99.91	100	100	*			
28	Skull Valley- Dermacentor parumapertus USA	99.91	99.96	99.96	99.96	99.96	99.82	99.96	99.82	99.91	99.82	99.82	99.96	99.87	99.82	99.91	99.91	99.78	99.78	99.78	99.78	99.78	99.78	99.96	99.96	99.96	99.96	99.96	*		
29	OSU 85-389- Dermacentor variabilis USA	99.87	99.91	99.91	99.91	99.91	99.78	99.91	99.87	99.87	99.87	99.87	99.91	99.82	99.78	99.87	99.87	99.74	99.74	99.74	99.74	99.74	99.74	100	99.91	100	100	100	99.96	*	
30	CA-459- Haemaphysalis leporispalustris USA	99.78	99.82	99.82	99.82	99.82	99.69	99.82	99.69	99.78	99.69	99.69	99.82	99.74	99.69	99.78	99.69	99.56	99.56	99.56	99.56	99.56	99.56	99.74	99.74	99.74	99.74	99.74	99.78	99.74	*
